# Calpain Inhibition Attenuates Adipose Tissue Inflammation and Fibrosis in Diet-induced Obese Mice

**DOI:** 10.1038/s41598-017-14719-9

**Published:** 2017-10-31

**Authors:** Latha Muniappan, Aida Javidan, Weihua Jiang, Shayan Mohammadmoradi, Jessica J. Moorleghen, Wendy S. Katz, Anju Balakrishnan, Deborah A. Howatt, Venkateswaran Subramanian

**Affiliations:** 1 Saha Cardiovascular Research Center, University of Kentucky, Lexington, KY USA; 2 Department of Physiology, University of Kentucky, Lexington, KY USA

## Abstract

Adipose tissue macrophages have been proposed as a link between obesity and insulin resistance. However, the mechanisms underlying these processes are not completely defined. Calpains are calcium-dependent neutral cysteine proteases that modulate cellular function and have been implicated in various inflammatory diseases. To define whether activated calpains influence diet-induced obesity and adipose tissue macrophage accumulation, mice that were either wild type (WT) or overexpressing calpastatin (CAST Tg), the endogenous inhibitor of calpains were fed with high (60% kcal) fat diet for 16 weeks. CAST overexpression did not influence high fat diet-induced body weight and fat mass gain throughout the study. Calpain inhibition showed a transient improvement in glucose tolerance at 5 weeks of HFD whereas it lost this effect on glucose and insulin tolerance at 16 weeks HFD in obese mice. However, CAST overexpression significantly reduced adipocyte apoptosis, adipose tissue collagen and macrophage accumulation as detected by TUNEL, Picro Sirius and F4/80 immunostaining, respectively. CAST overexpression significantly attenuated obesity-induced inflammatory responses in adipose tissue. Furthermore, calpain inhibition suppressed macrophage migration to adipose tissue *in vitro*. The present study demonstrates a pivotal role for calpains in mediating HFD-induced adipose tissue remodeling by influencing multiple functions including apoptosis, fibrosis and inflammation.

## Introduction

Accumulation of adipose tissue macrophages has been proposed as a major link between obesity, insulin resistance and type 2 diabetes^1^. However, the mechanisms underlying these processes are still remain elusive. Calpains are calcium dependent intracellular cysteine proteases that tightly regulate their substrate proteins through limited proteolysis^2^. The two major isoforms, calpain-1 and -2, are expressed ubiquitously, whereas the other isoforms (e.g. -3, -9) are tissue-specific^2^. Activated calpain by calcium damages cells by selectively degrading intracellular proteins, including signaling proteins (e.g., cyclin-dependent kinase, protein kinase C)^3,4^, cytoskeletal proteins (e.g., talin, spectrin)^5,6^, and transcription factors (e.g., c-Jun, IkB)^7–9^. Calpains play a critical role in cellular apoptosis through activation of both caspase-dependent and caspase-independent pathways^10,11^. Calpains are also involved in acute inflammatory processes via the activation of nuclear factor kappa B (NF-kB)^12^. Since calcium-induced calpain activation is an irreversible reaction, calpains are tightly regulated by calpastatin (CAST), which is an endogenous inhibitor that binds strongly to calpains^13^. CAST contains four tandem repeats of a calpain-inhibitory domain, and each CAST molecule is capable of inhibiting more than one calpain molecule^14^. Calpains have been implicated to play a critical deleterious role in endothelial dysfunction^15^, hypertrophy and fibrosis^16^, and intestinal macrophage activation in experimental colitis^17^.

Recently, using a pharmacological inhibitor and calpain-1 deficient mice, we demonstrated that calpain inhibition significantly attenuated inflammatory processes present in aortic vascular diseases such as angiotensin II (AngII)-induced abdominal aortic aneurysms (AAA) and atherosclerosis in mice^18,19^. The beneficial effect of calpain inhibition was associated with reduction of macrophage accumulation, NF-kB mediated inflammation, and cytoskeletal protein, filamin A, fragmentation in the aorta^19^. However, the functional contributions of calpain activation in chronic adipose tissue inflammation, and macrophage infiltration under the condition of obesity remains to be elucidated.

Using CAST transgenic mice, we demonstrate that calpain inhibition transiently improved glucose tolerance and resulted in decreased adipose tissue macrophage accumulation in obese mice. Furthermore, calpain inhibition significantly modulated adipose tissue remodeling by influencing multiple functions including adipocyte apoptosis, fibrosis, inflammation and migratory properties of macrophages.

## Results

### Calpain Protein and Activity Are Increased in Adipose Tissue of Obese Mice

**Figure 1:**
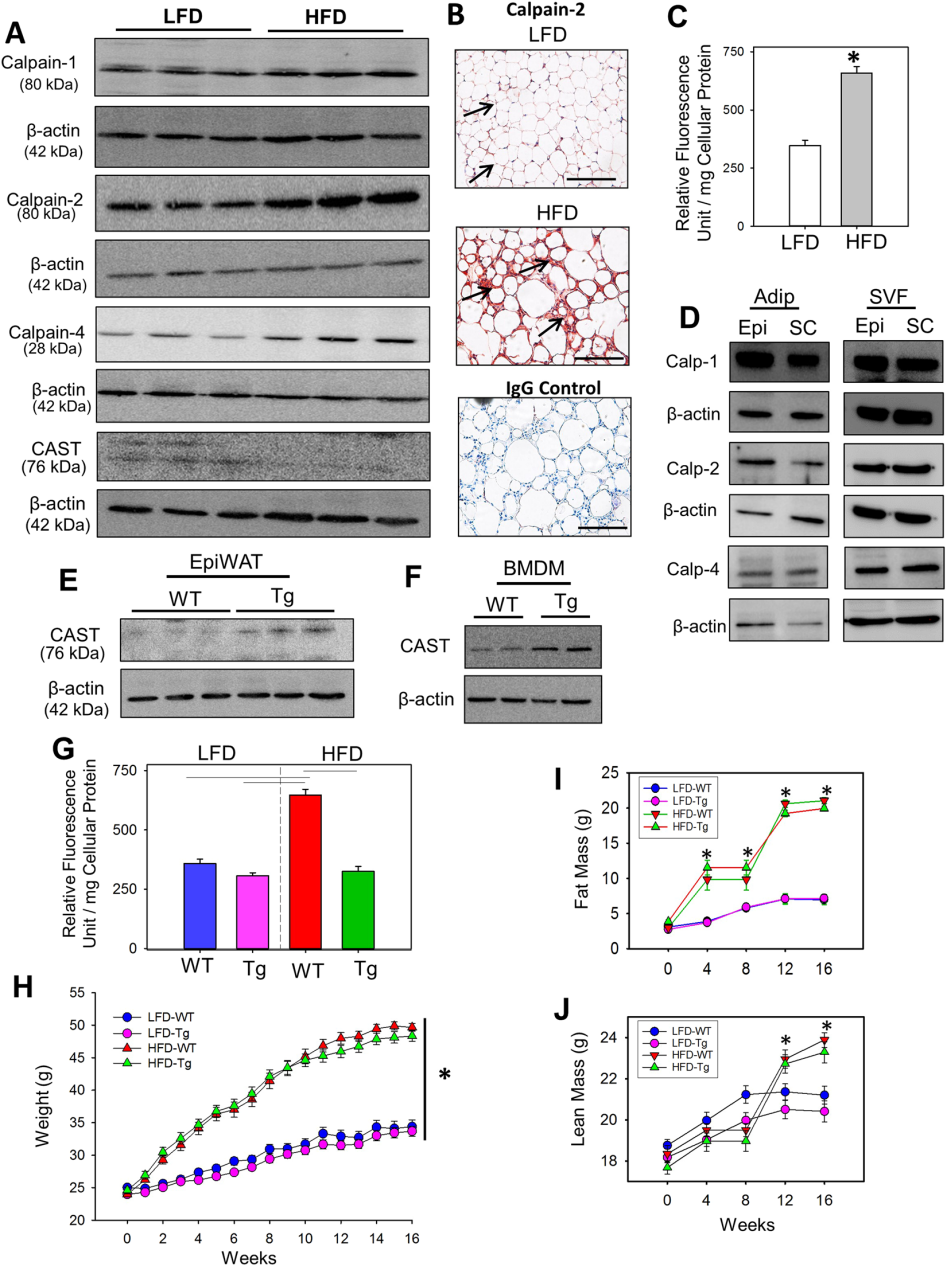
Abundance of calpain protein and its activity was increased in obese adipose tissue. (**A)** Calpain-1, -2, -4, CAST and β-actin protein were detected by Western blotting in epididymal white adipose tissue (EpiWAT) extracts of wild type mice fed either a LFD or HFD for 16 weeks (n = 3). (**B**) Cross-sections from the EpiWAT of LFD and HFD fed wild type mice were immunostained with anti-mouse calpain-2 (red color). Arrows indicate positive immunostaining. (**C**) Calpain activity was measured by fluorimetric assay in EpiWAT from LFD and HFD fed mice (n = 4). (**D**) Calpain-1, -2 and -4 protein were detected by Western blotting in matured adipocyte and SVF cells. CAST protein was detected in EpiWAT (**E**) and BMDM. (**F**) Representative Western blot images are cropped from the full-length blot. The full-length Western blots are included in the Supplementary information. (**G)** Calpain activity was measured by fluorimetric assay in EpiWAT from LFD and HFD fed WT and CAST Tg mice (n = 4). (**H**) Body weight of LFD and HFD fed male WT and CAST Tg mice (n = 13–15). Body fat mass (**I**) and lean mass (**J**) of LFD and HFD fed male WT and CAST Tg mice (n = 12–14). *And horizontal lines represent significance of *P* < 0.05 (Two-way ANOVA with Holm-Sidak post hoc analysis).

To determine whether obesity increases calpain protein and activity in adipose tissue, we harvested protein from adipose tissue of C57BL/6 mice fed a low fat diet (LFD; 10% Kcal) or high fat diet (HFD; 60% Kcal) for 16 weeks. Western blot analyses using antibodies against calpains showed that protein abundance of calpain-1 was slightly increased whereas the protein abundance of calpain-2 and calpain-4 (the common small subunit of both calpain-1 and -2) were highly increased in epididymal white adipose tissue (EpiWAT) from HFD fed obese mice compared to LFD fed lean mice (Fig. 1A). In addition, protein abundance of CAST was decreased in WAT from HFD fed obese mice (Fig. 1A). Furthermore, immunohistochemical staining of adipose tissue also confirmed increased calpain-2 protein in HFD mice compared to LFD lean mice (Fig. 1B). HFD-induced obesity resulted in a ~2.0 -fold increase in total calpain activity, as demonstrated by increased breakdown of fluorescent-labeled calpain substrate, Ac-LLY-AFC, in visceral adipose tissue (Fig. 1C). To delineate the cell type expressing calpain in adipose tissue, mature adipocytes and stromal vascular fractions (SVF) were harvested from adipose tissue of C57BL/6 mice. Western blot analyses using protein harvested from mature adipocytes and SVF-derived cells showed that calpain proteins are strongly expressed in both mature adipocytes and SVF (Fig. 1D).

### CAST Overexpression Had No Effect on Diet-induced Body Weight Gain and Fat Mass

To investigate the role of calpains in diet-induced obesity, we used CAST overexpressing transgenic mice to inhibit activities of both calpain-1 and -2. We confirmed presence of the CAST transgene (CAST-Tg) by PCR using tail DNA (Figure S1 in the online-only Data Supplement). Western blot analyses showed a modest ~ 2 fold increase in the abundance of CAST protein in EpiWAT (Fig. 1E) and bone marrow-derived macrophages (BMDM; Fig. 1F) of CAST-Tg mice compared to WT mice. This data confirms transgenic overexpression of CAST in different tissues.

To examine the role of calpains in diet induced obesity, male WT or CAST-Tg mice were fed with LFD or HFD for 16 weeks. HFD feeding resulted in a 2.2-fold increase in calpain activity, as demonstrated by increased breakdown of fluorescent-labeled calpain substrate, Ac-LLY-AFC, in EpiWAT harvested from WT mice, whereas CAST overexpression significantly attenuated HFD-induced calpain activity in EpiWAT (Fig. 1G). This data clearly suggests a strong inhibition of calpain activity in WAT by CAST overexpression.

HFD feeding significantly increased body weight in both WT and Tg groups of mice compared to LFD groups. However, CAST overexpression did not affect diet-induced body weight gain (Fig. 1H). To examine whether there are differences in body fat mass (Fig. 1I) and lean mass (Fig. 1J), we analyzed body composition before and after feeding HFD. Fat and lean mass were significantly increased in HFD mice of both WT and Tg groups compared with LFD controls, with no significant differences between genotypes. The mass of liver, epididymal, retroperitoneal, subcutaneous and brown adipose tissues were increased significantly in HFD fed mice of each genotype compared with LFD-fed controls, with no significant differences between the genotypes (Table 1). HFD feeding or CAST overexpression had no effect on blood cell counts (Table 1). CAST overexpression had no effect on food and water intake (Supplementary Table 1). HFD feeding increased adipocyte size in tissue sections from mice of each genotype. Quantification of adipocyte size demonstrated that there is no significant difference in adipocyte size distribution between the genotypes (Figure SII in the online-only Data Supplement). Therefore, CAST overexpression had no effect on the development of diet-induced obesity in mice fed HFD.

**Table 1:** Effects of calpastatin overexpression on liver, fat pad weight, and blood counts in LFD and HFD fed mice.

**Groups**	**CAST WT**		**CAST Tg**	
**Diet**	**LFD**	**HFD**	**LFD**	**HFD**
N	10	12	10	13
Liver Weight (g)	1.4 ± 0.06	2.8 ± 0.14*	1.3 ± 0.05	2.3 ± 0.16*
Epididymal Adipose (g)	1.0 ± 0.13	1.7 ± 0.09*	0.96 ± 0.06	1.5 ± 0.07*
Retroperitoneal Adipose (g)	0.3 ± 0.04	1.0 ± 0.03*	0.5 ± 0.04	1.2 ± 0.06*
Sub-cutaneous Adipose (g)	0.4 ± 0.05	2.4 ± 0.11*	0.4 ± 0.03	2.0 ± 0.12*
Brown Adipose (g)	0.3 ± 0.02	0.5 ± 0.03*	0.3 ± 0.02	0.4 ± 0.02*
RBC (10^6^/µl)	8.8 ± 0.5	11.7 ± 0.1	8.6 ± 0.5	10.5 ± 0.5
Platelets (10^3^/µl)	617 ± 29	514 ± 19	691 ± 49	587 ± 39

Values are represented as means ± SEMs. *Denotes *P* < 0.05 when comparing LFD vs HFD, by two-way ANOVA.

### CAST Overexpression Improved Glucose Tolerance in Early but not Late Stages of Obesity

**Figure 2:**
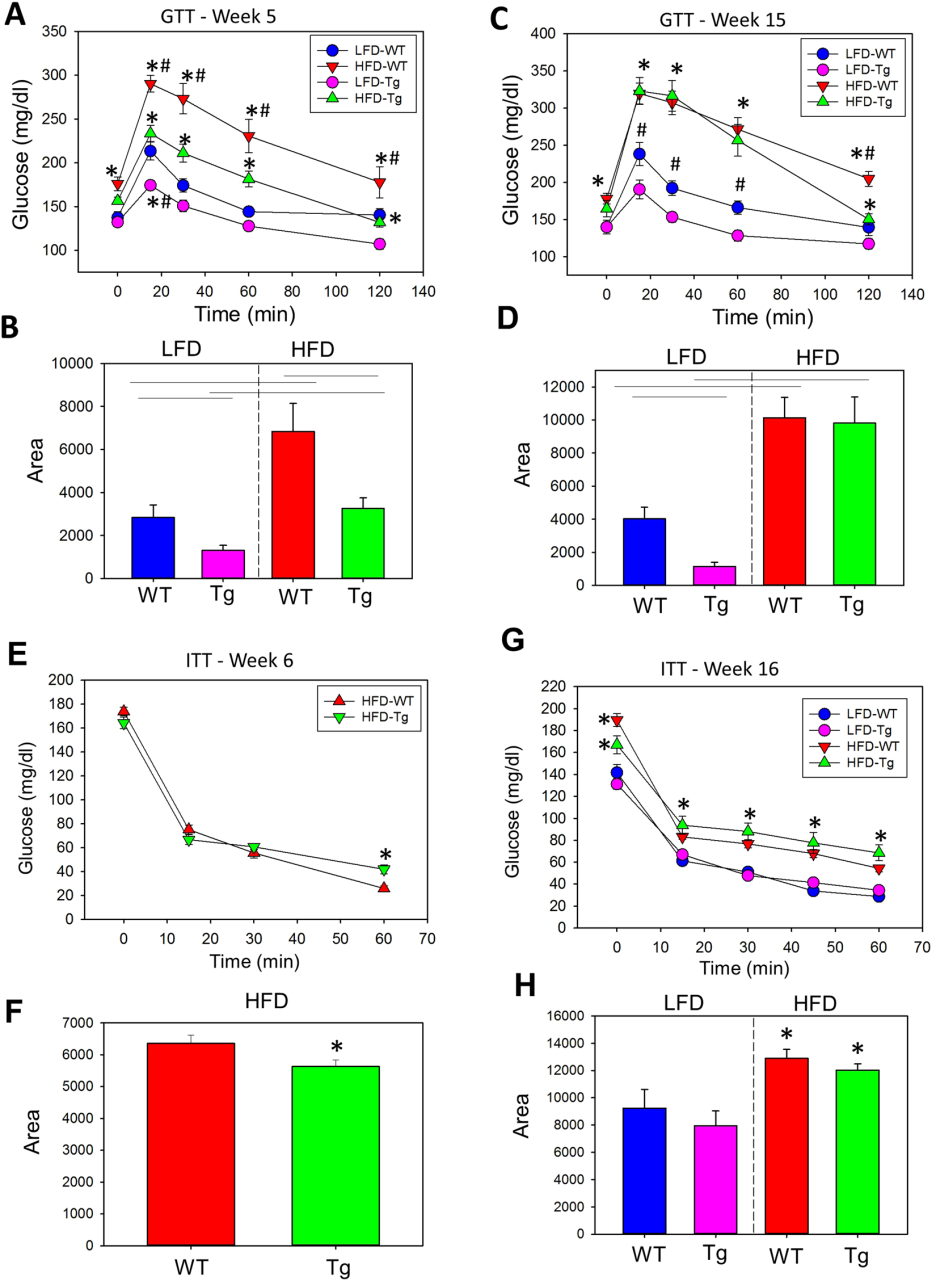
CAST overexpression improved glucose tolerance in the early stage of obesity. Glucose tolerance test (GTT) and the area under curve (AUC) of GTT at week 5 (**A,B**) and week 15 (**C,D**) post LFD and HFD in CAST WT and Tg mice. Insulin tolerance test (ITT) and the AUC of ITT at week 6 (**E,F**) and week 16 (**G,H**) post LFD and HFD in CAST WT and Tg mice (n = 12–14). *Denotes P < 0.05 HFD vs LFD; #P < 0.05 CAST WT vs CAST Tg (Two-way ANOVA with Holm-Sidak post hoc analysis).

To examine the effect of CAST overexpression on HFD-induced glucose tolerance, we performed glucose tolerance test (GTT) and insulin tolerance test (ITT) during an early (5 or 6 weeks post diet) and late (15 or 16 weeks post diet) stage of obesity. After 5 weeks of diet consumption, CAST Tg mice showed a dramatic improvement in glucose tolerance in both LFD and HFD groups (Fig. 2A,B) compared to the WT group (P < 0.05). While after 15 weeks of diet consumption, the CAST Tg HFD group showed no difference compared to the WT group; however the CAST Tg LFD group showed significant improvement compared to WT-LFD group (P < 0.05; Fig. 2C,D). With respect to ITT, HFD feeding significantly impaired insulin tolerance tests in both genotypes. However, CAST Tg mice showed a very modest improvement in ITT compared to WT group (Fig. 2E–H). Plasma insulin and leptin concentrations were increased significantly by HFD feeding in both genotype groups of mice (Figure SIII in the online-only Data Supplement). However, CAST Tg mice had a significant increase in plasma insulin concentration compared to WT-HFD group (Figure SIIIA,B). Irrespective of increased insulin concentration, CAST Tg mice showed no significant improvement on GTT. Furthermore, Western blot analyses of adipose tissue from either 5 or 16 weeks LFD and HFD mice showed no alterations in protein abundance of calpain-10, an atypical calpain associated with insulin resistance in diabetic populations^20,21^ (Figure SIIIE,F in the online-only Data Supplement).

### CAST Overexpression Suppressed High Fat Diet-induced Adipocyte Cell Death

**Figure 3:**
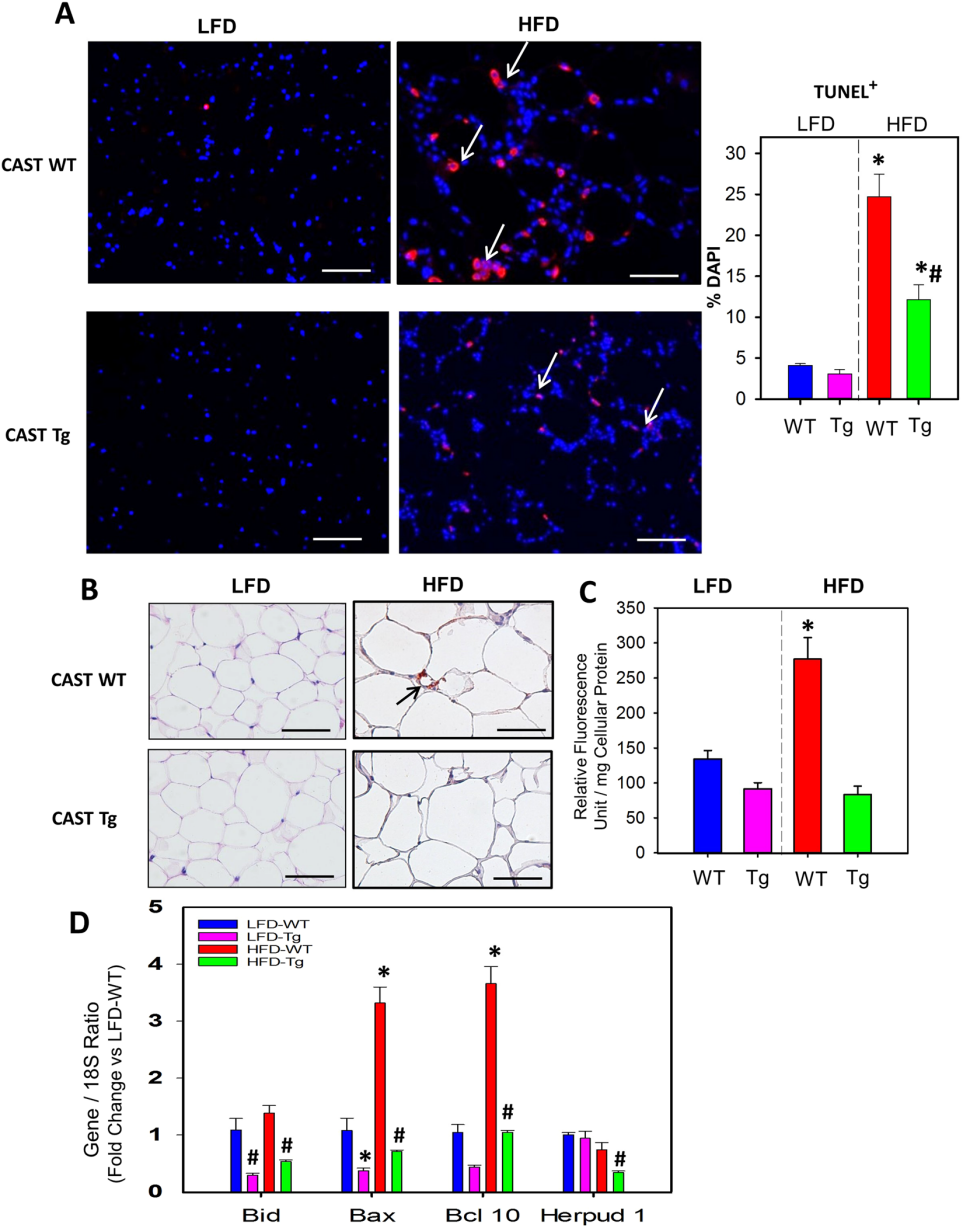
CAST overexpression significantly suppressed obesity-induced adipocyte cell death. (**A)** Representative TUNEL staining of EpiWAT cross-sections from 16 week LFD and HFD fed CAST WT and Tg mice. The nuclei were stained with DAPI (blue) and the TUNEL-positive cells (red) are indicated by arrows. Under fluorescent microscopy, TUNEL-positive cells were counted from 10 fields at the power of 100x magnification (n = 5). (**B**) Representative EpiWAT sections from LFD and HFD fed CAST WT and Tg mice immunostained for active caspase 3. Arrows indicate positive staining (red). (**C**) Caspase-3 activity was measured by fluorimetric assay in EpiWAT from LFD and HFD fed mice (n = 4). (**D**) mRNA abundance of Bid, Bax, Bcl 10 and Herpud genes in EpiWAT from LFD and HFD fed CAST WT and Tg mice were analyzed by qPCR (n = 4–6). Values are represented as mean ± SEM. *and # denotes *P* < 0.05 when comparing LFD vs HFD and WT vs Tg respectively (Two-way ANOVA with Holm-Sidak post hoc analysis).

Calpains play a critical role in calcium mediated cell apoptosis in association with caspase-3 pathway in various cell types^22,23^. Obesity is associated with increased adipocyte death, which in turn triggers macrophage accumulation for the removal of cell debris by phagocytosis^24,25^. To examine whether CAST overexpression had any influence on HFD-induced adipocyte cell death, we performed TUNEL staining on EpiWAT from 5 and 16 weeks LFD and HFD-fed groups of mice^26,27^. TUNEL staining showed very few TUNEL-positive (apoptotic) cells at 5 weeks of HFD feeding which is not statistically significant compared to LFD group (Figure SIV in the online-only Data Supplement). HFD feeding to mice for 16 weeks showed a significantly increased number of TUNEL-positive cells compared to LFD group (P < 0.05; Fig. 3A).

Compared to the WT group, CAST Tg overexpression showed significantly less number of TUNEL + cells (P < 0.05; Fig. 3A). Furthermore, immunostaining using an antibody against cleaved caspase-3, a marker of apoptosis^26^, showed increased staining in adipose tissue from WT mice, not in CAST Tg mice fed HFD (Fig. 3B). In addition, a fluorescent based caspase-3 activity assay also revealed a significant suppression of HFD-induced caspase-3 activity in CAST Tg mice compared to WT-HFD mice (P < 0.05; Fig. 3C). Furthermore, mRNA abundance of pro-apoptotic genes (e.g. Bax, BCL10) in adipose tissue were significantly increased at 16 week (Fig. 3D), but not in 5 week (Figure SIV in the online-only Data Supplement) HFD fed WT mice. In contrast, CAST overexpression significantly suppressed pro-apoptotic gene expression (P < 0.05; Fig. 3D) in 16 week HFD fed mice.

### CAST Overexpression Suppressed Adipose Tissue Macrophage Accumulation in Obese Mice

**Figure 4:**
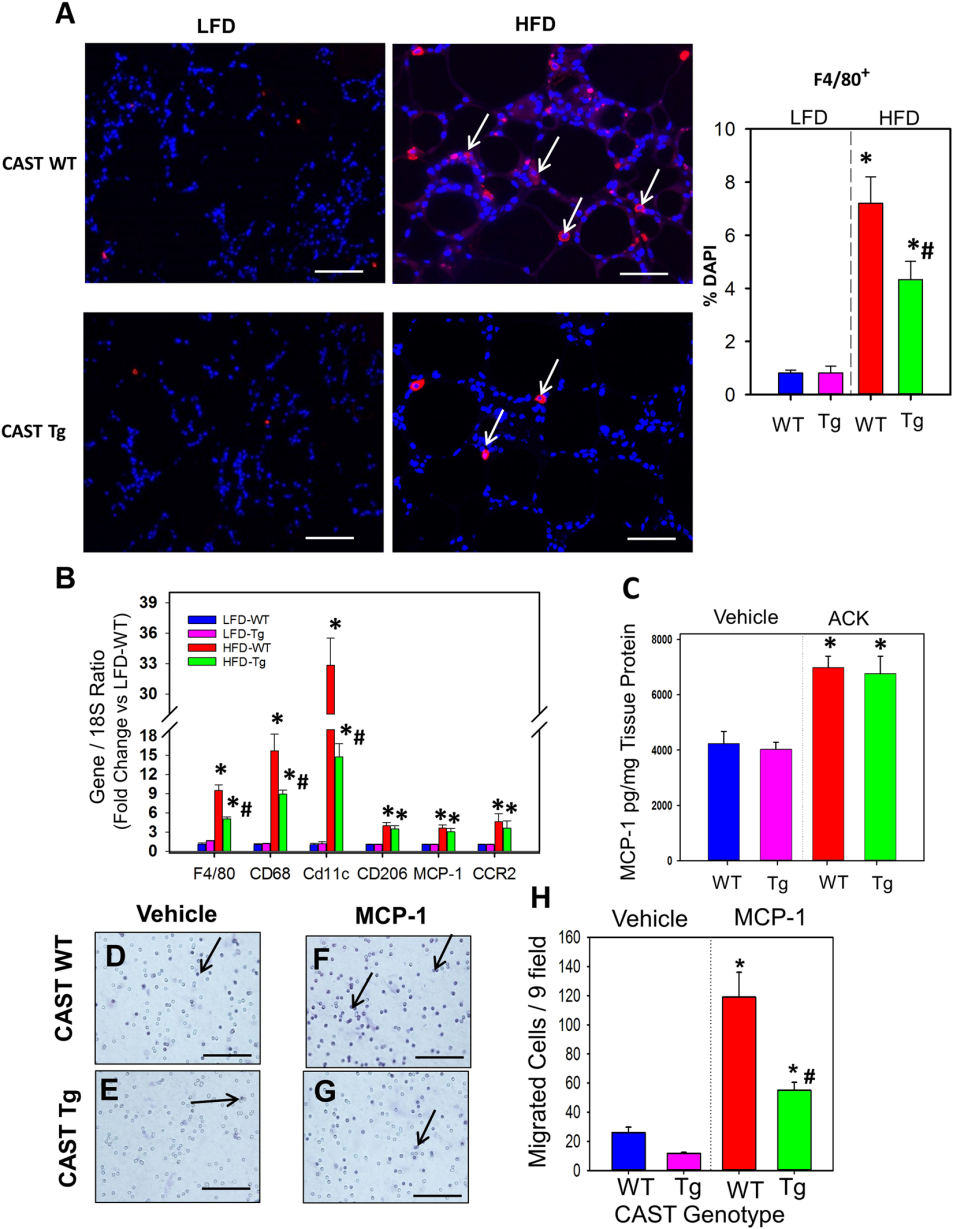
CAST overexpression significantly reduced macrophage accumulation. (**A**) Representative immunofluorescent staining of F4/80 in EpiWAT cross-sections from 16 week LFD and HFD fed CAST WT and Tg mice. The nuclei were stained with DAPI (blue) and the F4/80-positive cells (red) are indicated by arrows. Using fluorescent microscopy, F4/80-positive cells were counted from 10 fields at the power of 100x magnification (n = 5). (**B**) mRNA abundance of F4/80, CD68, CD11c, CD206, MCP-1 and CCR2 genes in EpiWAT from LFD and HFD fed CAST WT and Tg mice were analyzed by qPCR (n = 5). (**C)** MCP-1 protein accumulation in adipose tissue explant culture media was measured by ELISA. (**D-G)** WT and CAST-Tg BMDMs were seeded on transwell filters and lower chambers were filled with media containing either vehicle or MCP-1 (100 µg/mL). Cells that migrated through the membrane stained with hematoxylin and were counted from 9 fields at the power of 200x magnification (**H**) (n = 4). Values are represented as mean ± SEM. *and # denotes *P* < 0.05 when comparing LFD vs HFD and WT vs Tg respectively (Two-way ANOVA with Holm-Sidak post hoc analysis).

Since adipocyte apoptosis has been implied as one of the key events that contributes to adipose tissue macrophage infiltration^25^, we examined the contribution of calpains on macrophage accumulation during HFD-induced obesity. Immunofluorescent staining using an anti-F4/80 antibody revealed accumulation of F4/80 + macrophages in 5 and 16 weeks HFD-induced obese adipose tissue (Figure SV in the online-only Data Supplement and Fig. 4A). Compared to WT, CAST Tg overexpression had significantly less macrophage recruitment (Figure SVA and 4 A). Consistently, mRNA abundance of macrophage markers (F4/80, CD68, CD11C, CD206), monocyte chemoattractant protein-1 (MCP-1) and its cognate receptor, CCR2 were significantly increased in obese adipose tissue (Figure SVB and Fig. 4B) in HFD fed WT mice. In contrast, CAST Tg overexpression significantly reduced macrophage marker genes, but had no effect on MCP-1 and CCR2 (Fig. 4B). Since CAST overexpression had no effect on MCP-1 and CCR2 mRNA abundance, we examined whether CAST overexpression had any effect on MCP-1 secretion from adipose tissue. EpiWAT explants from WT and CAST Tg mice were incubated with adipogenic cocktail for 24 h^28^. Adipogenic cocktail incubation significantly increased MCP-1 concentrations in cultured media of adipose explants from both WT and CAST Tg mice (Fig. 4C). To define potential mechanisms by which CAST overexpression influenced macrophage accumulation in adipose tissue, we investigated whether CAST overexpression influenced functional properties of macrophages such as migration. Using bone marrow-derived macrophages (BMDMs) harvested from WT and CAST Tg mice, we examined the effect of CAST overexpression on macrophage migration towards monocyte chemoattractant, MCP-1, using an *in vitro* transwell migration assay^29^. MCP-1 strongly stimulated migration of macrophages from WT mice compared to vehicle control, whereas overexpression of CAST significantly reduced MCP-1-induced macrophage migration (Fig. 4D–H). These data suggest that calpain inhibition in macrophages by CAST overexpression decreased the migration abilities of macrophages towards adipose tissue.

### CAST Overexpression Attenuated HFD-induced Inflammation

**Figure 5:**
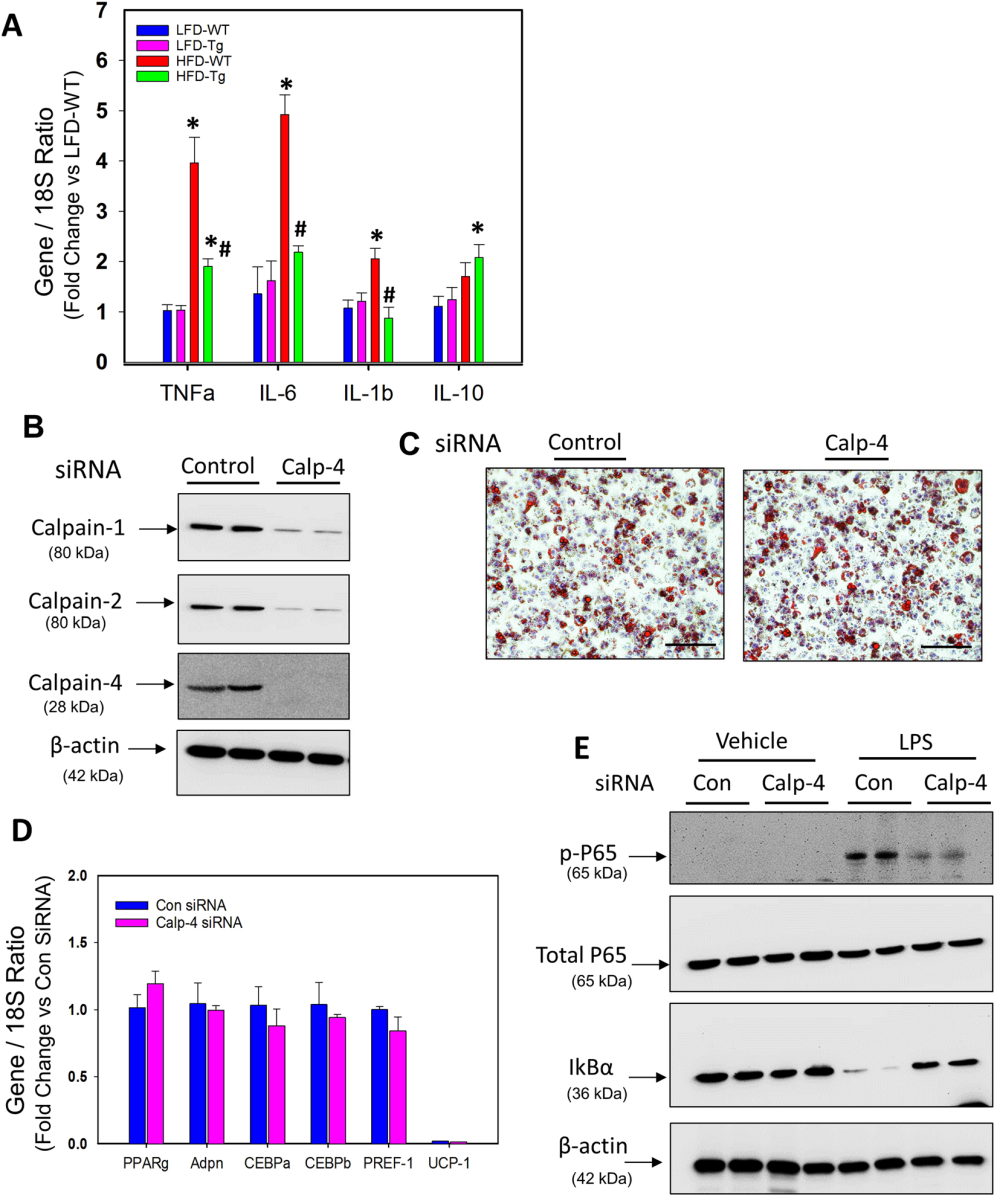
CAST overexpression or calpain-4 silencing significantly reduced inflammatory genes in EpiWAT tissue or cultured differentiated adipocytes. A. mRNA abundance of TNFα, IL-6, IL-1β, and IL-10 genes in EpiWAT from LFD and HFD fed CAST WT and Tg mice were analyzed by qPCR (n = 5). (**B**) Western blot analyses of Calpain-1, -2, -4 and β-actin proteins in control or calpain-4 siRNA transfected differentiated 3T3L1 adipocytes. (**C**) Oil red O staining of control or calpain-4 siRNA transfected differentiated 3T3L1 adipocytes. D. mRNA abundance of PPARγ, adiponectin, CEBPα, CEBPβ, PREF-1 and UCP-1 genes in control or calpain-4 siRNA transfected differentiated 3T3L1 adipocytes were analyzed by qPCR (n = 5). (**E**) Western blot analyses of phospho and total P65, IkBα and β-actin proteins in control or calpain-4 siRNA transfected differentiated 3T3L1 adipocytes incubated with vehicle or LPS (100ng/ml for 24 h; n = 4). Representative Western blot images are cropped from the full-length blot. The full-length Western blots are included in the Supplementary information. Values are represented as mean ± SEM. *and # denotes *P* < 0.05 when comparing LFD vs HFD and WT vs Tg respectively (Two-way ANOVA with Holm-Sidak post hoc analysis).

To further investigate the role of calpain on HFD-induced adipose tissue inflammation, mRNA abundance of inflammatory cytokines such as TNFα, IL-6, IL-1β, IL-10) were examined in EpiWAT tissue. HFD-induced obesity significantly increased mRNA abundance of TNFα and IL-6 in both 5 (Figure SVC in the online-only Data Supplement) and 16 week points, IL-1β at 16 weeks (Fig. 5A), and had no effect on IL-10 (Fig. 5A). In contrast, CAST overexpression significantly reduced HFD-induced TNFα, IL-6 and IL-1β gene expression (Figure SVC and Fig. 5A).

IKK-dependent NF-kB activation has been implicated in the development of obesity^30^. Activated NF-kB is also shown to induce inflammatory cytokines such as IL-6 and TNF-α^31^. NF-kB is sequestered in the cytosol and its translocation to the nucleus is prevented by the inhibitor of NF-kB translocation (IkB)^32^. IκB was also shown to be a target of calpain in selected cell types including macrophages^17,33,34^. In order to investigate the possibility that calpain activation mediates obesity-induced NF-kB activation, we used 3T3-L1 adipocytes as a model system. Undifferentiated and differentiated 3T3-L1 adipocytes were transfected with control or calpain-4 (common small subunit of calpain-1 and -2) siRNA for 48 h. The cells were then stimulated with lipopolysaccharide (LPS) for 24 h. Protein was extracted from the cells and subjected to Western blot analyses. Transfection of cells with calpain-4 siRNA strongly suppressed calpain-1 and -2 protein as evidenced by Western blot analyses (Fig. 5B). To delineate whether calpain-4 silencing influences adipogenesis, Oil red O staining was performed on siRNA transfected cells. Oil red O staining showed that calpain-4 silencing had no effect on adipocyte differentiation (Fig. 5C). Furthermore, qPCR analyses showed comparable mRNA abundance of adipogenic genes such as PPARγ, CEPBα, CEBPβ, PREF-1 and adiponectin (Fig. 5D). NF-kB activation and IkB degradation were assessed by examining the phosphorylation of NF-kB subunit P65, and IkB protein by Western blot. LPS stimulation significantly reduced IkB protein and significantly increased phosphorylation of P65 in control siRNA transfected cells (Fig. 5E). In contrast, silencing of calpain-1 and -2 significantly prevented LPS-induced IkB protein degradation and P65 phosphorylation in differentiated adipocytes (Fig. 5E).

### CAST Overexpression Suppressed HFD-induced Adipose Tissue Fibrosis

**Figure 6:**
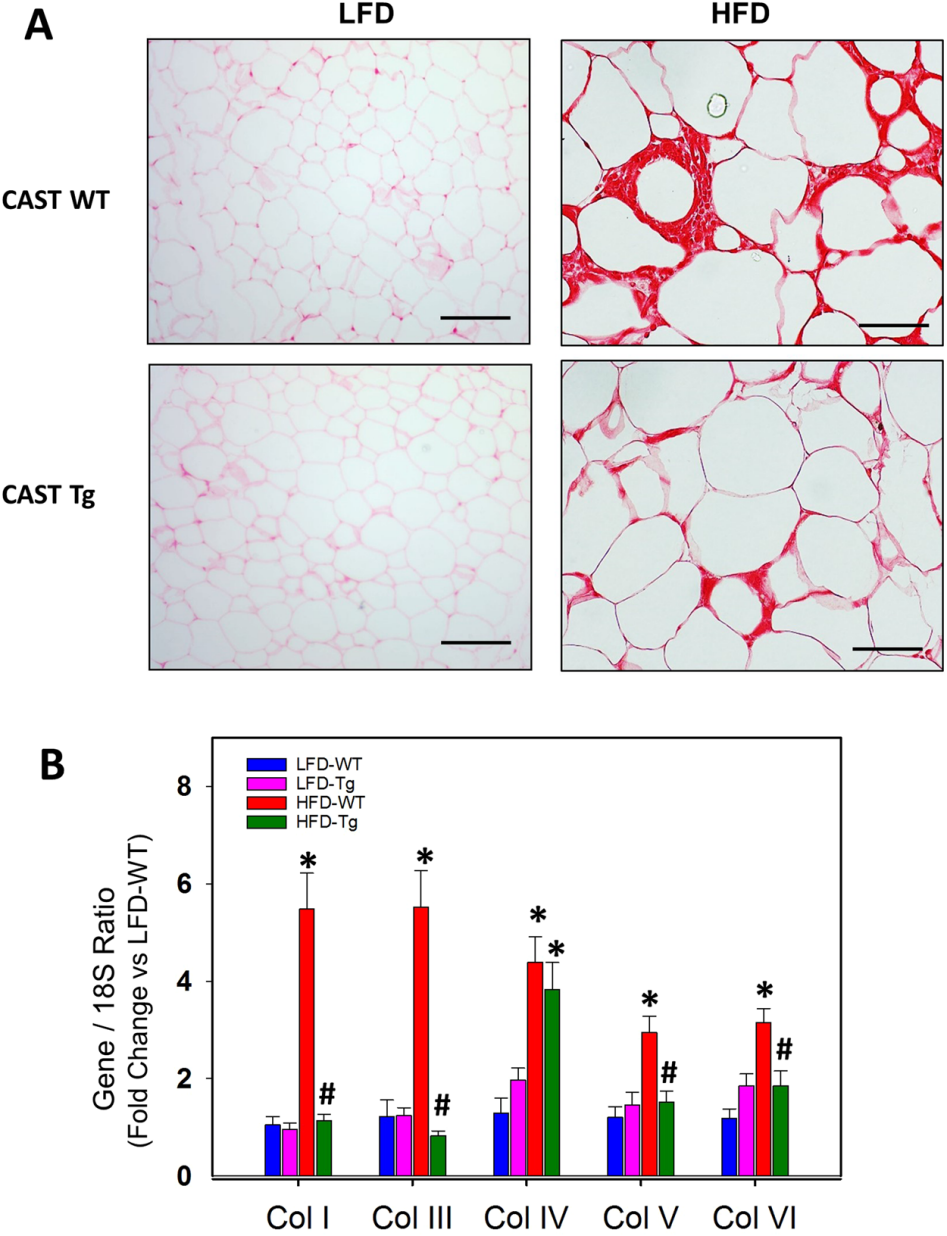
CAST overexpression reduced interstitial fibrosis in adipose tissue. (**A**) Representative Sirius red staining of EpiWAT cross-sections from LFD and HFD fed CAST WT and Tg mice. (**B**) mRNA abundance of Col I, Col III, Col IV, Col V and Col VI genes in EpiWAT from LFD and HFD fed CAST WT and Tg mice were analyzed by qPCR (n = 5). Values are represented as mean ± SEM. *and # denotes *P* < 0.05 when comparing LFD vs HFD and WT vs Tg respectively (Two-way ANOVA with Holm-Sidak post hoc analysis).

Recently, excessive amounts of extracellular matrix and fibrosis in white adipose tissue have been described in obese individuals^35^. Increased WAT fibrosis is believed to accelerate adipose tissue dysfunction^36^. In order to understand the effect of CAST overexpression on WAT fibrosis, we performed picro-sirius red staining on EpiWAT sections from 5 week (Figure SVI in the online-only Data Supplement) and 16 week (Fig. 6A) LFD and HFD fed mice. HFD feeding for 16 weeks but not 5 weeks increased adipose tissue fibrosis compared to the LFD group. In contrast, CAST overexpression dramatically suppressed collagen deposition in the WAT tissue compared to WT group of mice (Fig. 6A). Furthermore, qPCR analyses of WAT mRNA showed that CAST overexpression significantly suppressed HFD-induced collagen genes (I, III, V and VI) compared to WT group (Fig. 6B).

### CAST Overexpression Had no Effect on HFD-induced Liver Steatosis

**Figure 7:**
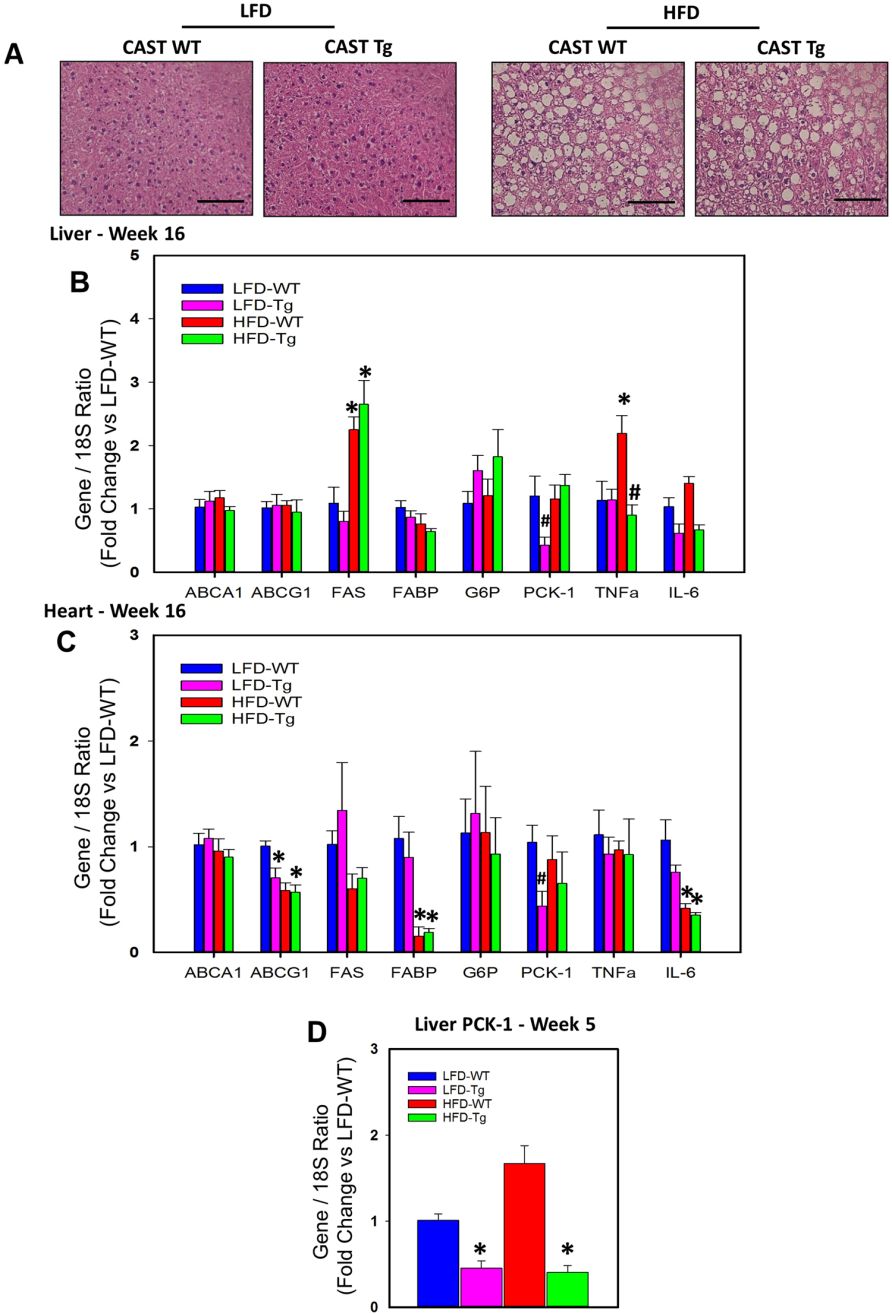
CAST overexpression had no effect on obesity-induced hepatic steatosis. (**A**) Representative hematoxylin and eosin staining of liver cross-sections from LFD and HFD fed CAST WT and Tg mice. mRNA abundance of ABCA1, ABCG1, FAS, FABP, G6P, PCK-1, TNF-α and IL-6 genes in liver (**B**) and heart (**C**) from 16 weeks LFD and HFD fed CAST WT and Tg mice, and PCK-1 gene in liver (**D**) from 5 week LFD and HFD fed CAST WT and Tg mice were analyzed by qPCR (n = 5). Values are represented as mean ± SEM. *and # denotes *P* < 0.05 when comparing LFD vs HFD and WT vs Tg respectively (Two-way ANOVA with Holm-Sidak post hoc analysis).

Since CAST overexpression suppressed HFD-induced adipose tissue inflammation, macrophage accumulation and apoptosis without influencing glucose clearance, next we examined whether CAST overexpression had any effect on other metabolic organs such as liver and heart in HFD-induced obese mice. Histological analyses showed similar lipid deposition in liver tissue from both WT and CAST Tg mice fed with HFD diet compared to LFD fed mice (Fig. 7A). In addition, qPCR analyses of selected genes using mRNA from liver and heart tissue showed comparable alterations in both CAST WT and Tg groups (Fig. 7B,C). HFD feeding significantly increased fatty acid synthase (FAS) gene expression whereas it had no significant effect on fatty acid binding protein (FABP), lipid transporters (ABCA1, ABCG1) in liver. CAST Tg overexpression had no effect on HFD-induced FAS in liver (Fig. 7B). In heart tissue, HFD feeding significantly suppressed FABP and ABCG1, whereas CAST Tg overexpression had no effect on these HFD-induced gene alterations (Fig. 7C). With respect to inflammation, CAST Tg overexpression significantly suppressed HFD-induced TNFα gene expression in liver (Fig. 7B).

### CAST Overexpression Transiently Suppressed Phosphoenolpyruvate Carboxykinase 1 (PCK-1) Gene Expression in Liver and Heart Tissue of HFD Fed Obese Mice

qPCR analyses of liver and heart tissue showed that HFD feeding for 16 weeks had no effect on glucose metabolic genes such as glucose-6-phosphatase (G6P) and phosphoenolpyruvate carboxykinase 1 (PCK-1). Interestingly, CAST Tg overexpression showed a significant reduction on PCK-1 mRNA abundance in LFD fed mice compared to WT (Fig. 7B, C). Suppression of PCK-1 in liver is shown to promote insulin sensitivity in obese mice^37^. Coinciding with these observations, the glucose tolerance test studies also showed a significant improvement on glucose clearance at 16 weeks in LFD fed CAST Tg mice compared to other groups (Fig. 2C). In addition, overexpression of PCK-1 in adipose tissue is shown to cause insulin resistance in HFD fed mice^38^. However, qPCR analyses of adipose tissue revealed that CAST Tg overexpression had no influence on adipose PCK-1 (Figure SVII in the online-only Data Supplement). Since CAST Tg overexpression showed a transient improvement in glucose clearance (5 weeks post HFD), next we tested the effect of CAST Tg overexpression on PCK-1 in adipose (Figure SVII in the online-only Data Supplement) and liver (Fig. 7D) tissue from 5 weeks LFD and HFD mice by qPCR. CAST Tg overexpression had no effect on adipose PCK-1 mRNA (SVIIB), whereas it showed a strong suppression of liver PCK-1 in both LFD and HFD group compared to WT controls (Fig. 7D).

## Discussion

During the course of obesity, adipose tissue undergoes extensive remodeling including adipocyte hypertrophy, apoptosis, macrophage infiltration and accumulation, and fibrosis. Using CAST transgenic mice, we examined the role of calpains in adipose tissue remodeling during diet induced-obesity. Here we demonstrate that calpain inhibition resulted in decreased adipose tissue inflammation. The beneficial effect of calpain inhibition was associated with the reduction of macrophage accumulation and inflammation, which attributed to blunted functional properties of macrophage migration. Furthermore, calpain inhibition significantly modulated adipose tissue remodeling by influencing fibrosis and adipocyte apoptosis. This is the first report to document the functional role of calpain in adipose tissue remodeling during obesity.

In the present study, HFD-induced obesity in mice profoundly increased adipose tissue calpain protein and activity. CAST overexpression significantly suppressed HFD-induced calpain activity, as evidenced by the suppressed breakdown of fluorescent labeled calpain substrate. This result is consistent with our recently published and other earlier studies showing that CAST overexpression sufficiently suppressed high fat and AngII-induced calpain activity in various tissues^16,29^. Using a diet-induced obesity model, our current study revealed that CAST overexpression-mediated calpain inhibition had no effect on the development of obesity and did not affect food intake. Consistent with this current observation, several recent pathological studies from our group and others examined calpain function in mice fed high cholesterol or fat enriched diets and showed calpain deficiency or inhibition had no effect on body weight in mice^18,19,29,39^. However, in a recent study, administration of BDA-410, a non-selective calpain inhibitor, to chow fed 23-month old sedentary female mice induced loss of body weight and fat mass^40^. The loss in body weight was mainly due to muscle wasting and lipolysis^40^. The difference in body weight change between this published study and our current observation could be explained by the following factors: (i) Age - 10 weeks vs 23 months. (ii) The diet model - the HFD-induced body weight gain is more robust than chow diet. In addition, in our current study, calpain inhibition had no effect on body weight gain under either LFD or HFD condition. It appears that calpain inhibition may promote lipolysis under chow fed diet condition, whereas it had no effect on HFD-induced fat accumulation. (iii) The another important factor is sex. The BDA-410 study used only aged females whereas our current study is on males. It is not clear whether calpain plays a diverse role in male and female in regulating lipolysis and body weight gain. Further studies are required using both male and female genders side by side under 2 different diets (LFD and HFD) to examine whether the age and sex play a critical role in calpain mediated body weight regulation under different diet conditions.

Furthermore, GTT and ITT studies indicated that HFD feeding exacerbated obesity associated insulin resistance. Interestingly, CAST overexpression showed a slight and significant improvement in the early stage (5 weeks) of obesity, which disappeared in the late stage of obesity (16 weeks). At 5 weeks of diet-induced obesity, fasting plasma insulin concentration was significantly higher in HFD-CAST Tg mice compared to HFD-WT and LFD fed mice. At 16 weeks of diet-induced obesity, plasma insulin concentration was significantly higher in both HFD- WT and Tg mice (Figure SIII in the online-only Data Supplement). However, the magnitude of increase was significantly higher in Tg group compared to WT group. Interestingly, irrespective of greater insulin concentration, CAST Tg group showed no significant improvement on GTT compared to WT group. CAST is well known to inhibit both calpain-1 and -2 activity^41^. Calpain-10, a member of atypical calpains, is shown to be associated with type 2 diabetes and insulin resistance due to a variation in the calpain-10 gene in type 2 diabetes population with a single nucleotide polymorphisms at a rare allele SNP 44^20,21^. Calpain-10 lacks a penta-EF hand in domain IV of its catalytic subunit and are unable to bind to the CAST^2,42^. Our western blot studies using calpain-10 antibodies ruled out that there is no alteration in calpain-10 protein in EPiWAT of HFD-fed CAST Tg mice either at 5 or 16 weeks (Figure SIIIE,F in the online-only Data Supplement).

Interestingly, our qPCR analyses of liver tissue from 5 and 16 weeks LFD and HFD groups showed a strong suppression of PCK-1 gene in CAST Tg mice fed with LFD for 5 and 16 weeks. In the case of HFD feeding, CAST Tg overexpression showed a significant suppression of hepatic PCK-1 at 5 weeks, whereas the effect disappeared post 16 weeks HFD feeding. Suppression of PCK-1 in liver is shown to promote insulin sensitivity in obese mice^37^. Consistently, the GTT studies showed a significant improvement in glucose clearance in CAST Tg mice fed with HFD for 5 weeks. In addition, CAST Tg overexpression showed a significant improvement in glucose clearance in LFD fed mice post 16 weeks, whereas the beneficial effect of CAST Tg overexpression disappeared upon HFD feeding. In consistent, the PCK-1 mRNA expression was comparable between WT and Tg groups post 16 HFD feeding. In addition, the CAST Tg overexpression had no beneficial effect on GTT post 16 weeks. These data clearly indicate that CAST Tg mediated calpain inhibition transiently suppresses hepatic PCK-1, which in turn promotes insulin sensitivity in the early stage of obesity. Further studies are warranted to understand the mechanisms by which (i) calpain activation promotes PCK-1 gene expression and activity and (ii) CAST overexpression leads to insulin resistance in long term obesity.

Adipocyte cell death has been proposed as a key mechanism that leads to macrophage infiltration into adipose tissue during the development of obesity^24,25^. In support, genetic inactivation of pro-apoptotic gene, Bid in mice prevented adipocyte death and adipose tissue macrophage infiltration^26^. Calpains are shown to play a critical role in calcium mediated cell apoptosis in association with caspase-3 pathway in various cell types^22,23^. In consistent, we observed increased adipocyte death as evidenced by increased TUNEL positive nuclei and increased cleaved caspase activity in HFD-induced mice. CAST Tg overexpression mediated calpain inhibition significantly suppressed obesity induced apoptotic cell death, pro-apoptotic genes (Bid, Bax, and Bcl10), and adipose tissue macrophage accumulation. With respect to adipose tissue macrophage accumulation, CAST overexpression had no influence on adipose tissue MCP-1 secretion, a major monocyte chemoattractant protein. In contrast, CAST overexpression impaired macrophage migration property towards MCP-1, which may have contributed to decreased macrophage accumulation in adipose tissue. In support, in our very recent published study, we showed that CAST overexpression in macrophages suppressed AngII-induced macrophage migration and adhesion to endothelial cells *in vitro* ^29^. In the present study, calpain inhibition reduced obesity-induced pro-inflammatory gene expression including TNFα, IL-6, and IL-1β in adipose tissue. In addition, calpain silencing in differentiated adipocytes suppressed LPS-induced NF-kB activation (P65 phosphorylation) and prevented LPS-induced IkB degradation. IKK-dependent NF-kB activation has been implicated in the development of obesity and adipose tissue inflammation^30,43^. NF-kB translocation inhibitor, IκB, was also shown to be a target of calpain in selected cell types including macrophages^17,32–34^. In support, CAST deficiency in intestinal macrophages also induced NF-kB translocation to the nucleus^17^. Furthermore, deletion of calpain-4, the common small subunit of calpain-1 and -2 in cardiomyocytes resulted in suppressed NF-kB activity followed myocardial infarction in mice^12^. NF-kB activation plays an important role in promoting expression of various proinflammatory factors (e.g. MCP-1, IL-6), which mediate inflammatory responses^44,45^. The observed reduction in adipocyte apoptosis, in addition to, impaired macrophage migration, and accumulation upon CAST overexpression may well correlate with the observed reduction of obesity-induced adipose tissue inflammation.

Overproduction of extracellular matrix components in adipose tissue has been implicated in systemic insulin resistance and hepatic steatosis in obese mice and humans^35,46,47^. In the present study, CAST overexpression strongly suppressed the induction of various collagen genes which are well known to play a critical role in the production of matrix components in adipose tissue. Recently, accumulated macrophages are also shown to play an important role in the regulation of adipose tissue fibrosis^48^. Consistent with these reports, we observed a strong reduction in macrophage accumulation in CAST overexpressing mice, which in turn may contribute to suppression of collagen gene induction in Tg mice adipose tissue. However, future studies are required to understand the mechanisms through which calpain regulates functional properties of macrophages in regulating matrix production and subsequent fibrosis.

In summary, we demonstrated that inhibition of calpain results in decreased obesity-induced adipose tissue inflammation, which is associated with reduced adipocyte apoptosis and fibrosis. These results suggest that inhibition of calpain activity may offer a new therapeutic target to reduce obesity-induced adipose tissue inflammation.

## Methods

### Mice

Mice overexpressing calpastatin (CAST-Tg) driven by a cytomegalovirus promoter on a C57BL/6 background were generated originally in the laboratory of Dr. Laurent Baud^49^. CAST-Tg mice were backcrossed at least 9 generations into a C57BL/6 background. C57BL/6 J (Stock# 000664) mice were purchased from The Jackson Laboratory (Bar Harbor, ME). Age-matched controls (8–10 weeks old) were used for the present study. Mice were maintained in a barrier facility. All study procedures were approved by the University of Kentucky Institutional Animal Care and Use Committee (Protocol # 2011–0907). This study followed the recommendations of The Guide for the Care and Use of Laboratory Animals (National Institutes of Health).

### Mouse Genotyping

Mouse genotypes were confirmed by PCR. DNA was isolated from tail snips or BM-derived cells using a Maxwell tissue DNA kit (Cat# AS1030, Promega, Madison, WI). CAST-Tg genotyping was performed using the following primers: 5′-GTTGGCTTAGGCTGCTTTTCGT-3′ and 5′-CCAGACTCCGTGA CACCCCTT-3′. The resultant CAST-Tg PCR product was 518 base pairs (bp) and no product for non-transgenic mice. The IL-2 gene was used as an internal control for CAST-Tg genotyping using the following primers: 5′-CTAGGCCACAGAATTGAAAGATCT and 5′-GTAGGTGGAAATTCTAGCATCATCC. The resultant product was 324 bp (Figure SI in the online-only Data Supplement).

### Diet

Mice were fed a low fat diet (LFD, 10% kcal as fat; D12450B; Research Diets Inc, New Brunswick, NJ) or high fat diet (HFD, 60% kcal as fat; D12492, Research Diets Inc, New Brunswick, NJ) for 5 or 16 weeks. Mice were provided water and diet *ad libitum*. At study endpoint, mice were anesthetized with a mixture of ketamine/xylazine (100/10 mg/kg, i.p) for exsanguination and tissue harvest. Blood was collected in tubes containing EDTA (0.2 mol/L), centrifuged at 2,000 rpm for 20 min (4 °C) and plasma was stored at -80 °C. Tissues were snap frozen in liquid nitrogen and stored at −80 °C.

### Metabolic and Histological Analyses

Body weight was measured weekly. At baseline and study endpoints, fat and lean mass were measured on conscious mice using NMR spectroscopy (Echo MRI). Intraperitoneal glucose tolerance test (GTT) and insulin tolerance test (ITT) were performed after 5 weeks or 15 weeks of LFD or HFD feeding. Mice were fasted either for 6 h (GTT) or 4 h (ITT) before intraperitoneal injections of glucose (2 g/kg body weight) or insulin (0.5 unit/kg body weight; Novolin R, Novo Nordisk Inc.). Blood glucose concentrations were measured using a glucometer at 0, 15, 30, 45, 60 and 120 minutes post injection. Histological, immunohistochemical and immunofluorescent staining of adipose tissue sections were performed on formalin-fixed sections, with appropriate negative controls, as described previously^50,51^. Immunohistochemical staining was performed on adipose sections to detect calpain, F4/80 positive macrophages and active caspase-3 protein. Calpain-2 immunostaining was performed using the rabbit anti-mouse calpain-2 (10 µg/ml, catalog No. RP-2 Calpain-2; Triple Point biologics, Forest Grove, OR). The F4/80 and active caspase-3 staining were performed using the following antibodies: rat anti-mouse F4/80 (1:200, catalog No. ab6640; Serotec, Abcam, Cambridge, MA) for macrophages; and rabbit anti-mouse cleaved caspase-3 (2 µg/ml, catalog No: 9661; Cell Signaling, Beverly, MA) for active caspase-3. TUNEL staining for adipose tissue sections was performed using the *IN Situ* Cell Death Detection Kit (Roche Applied Science).

### Adipocyte Size Analyses

Hematoxylin and eosin stained EpiWAT sections were imaged at 10x magnification and the size of adipocytes were quantified as described previously^52^. Briefly, using the measurement features of NIS Elements software including detection of edges, image thresholding, and object counting, the area and number of adipocytes within a 700 × 700 µm measurement frame were quantified. Adipocyte numbers were quantified on three measurement frames within each section of EpiWAT (n = 5 mice in each group).

### Measurement of Blood and Plasma Components

Peripheral blood cell numbers were counted using a Hemavet 950 (Drew Scientific Inc, Dallas, TX). Plasma insulin, leptin and adiponectin concentrations were measured using commercially available ELISA kits (R&D systems) as described previously^50^.

### Macrophage Migration Assay

Migration assays were performed using transwells with 8.0-μm pore polycarbonate membrane inserts (Corning)^53^. Bone marrow cells were harvested from the femurs of WT and CAST-Tg mice^54^ and differentiated into bone marrow-derived macrophages (BMDMs) using RPMI media containing 10% FBS and 15% L929 Cells (mouse fibroblast) conditioned medium for 7 days^29^. The fresh media was provided every 48 h. On day 7, WT and CAST-Tg BMDMs were trypsinized, counted, and seeded (1 × 10^6^ in a volume of 50 μl media/well) on transwell filters, and lower chambers were filled with either control media or media containing MCP-1 (100 ng/mL). After a 6 h incubation at 37 °C, cells were removed from the upper surface of inserts by scraping with Q-Tips. The membranes were fixed with 1% glutaraldehyde (Sigma; catalog No: G5882), stained with hematoxylin (Leica) and mounted on glass slides using glycerol gelatin. Hematoxylin-stained cells were counted using a microscope (Nikon, Melville, NY) by two independent investigators in a blinded manner. An example of the membrane stained with hematoxylin is shown in the supplement information (Figure SVIII in the online-only Data Supplement). Only the cells that migrated through the pores in response to MCP-1 are stained with hematoxylin. The non-hematoxylin stained circles are not cells, but rather transwell membrane pores. The hematoxylin stained cells were counted from 9 fields at the power of 200x magnification and quantified (n = 4).

### Quantification of MCP-1 Release from Adipose Tissue Explants by ELISA

Adipose tissue explants (epididymal fat, 50 mg) harvested from WT and CAST-Tg mice were incubated with vehicle or adipogenic cocktail (100 nM insulin, 1 µM dexamethasone, 0.5 mM IBMX) for 24 h in a 12 well plate. Culture media from tissue explants were collected and centrifuged at 13,000 rpm for 5 minutes. Supernatants were stored at −80 °C until assay. Accumulation of MCP-1 protein in media was measured with a mouse MCP-1 ELISA kit (R & D System; catalog No: MJE00) and normalized to tissue explant protein.

### 3T3-L1 Cell Differentiation and Short Interfering RNA Knockdown

3T3-L1 cells were first differentiated into mature adipocytes as described previously^55^. Differentiated 3T3-L1 cells were transfected with control short interfering (si)RNA or siRNA targeting the mouse calpain-4 (the common small subunit of calpain-1 and -2; Ambion - Silencer Select validated siRNA, Thermo Fisher Scientific) sequences using RNAiMax lipofectamine transfection reagent (Life Technologies, Thermo Fisher Scientific). After 48 h of transfection, cells were treated with vehicle or LPS (100ng/ml) for 24 h for western blot analyses.

### mRNA Abundance

Total RNA was extracted from adipose tissue or cells using the SV Total RNA Isolation System (Promega; catalog No: Z3100). RNA (100 ng) was reverse transcribed using the iScript cDNA synthesis kit (Cat #170–8891; Bio-Rad, Hercules, CA). Quantitative PCR was performed to quantify mRNA abundance using a SsoFas EvaGreen Supermix kit (Cat # 172–5203; Bio-Rad) on a Bio-Rad CFX96 cycler. mRNA abundances were calculated by normalization to internal control 18 S rRNA. Non-template and no RT reactions were used as negative controls. The primers used are detailed in Supplementary Table 2.

### Western Blot Analyses

Cell or tissue lysates were extracted in radio immunoprecipitation assay lysis buffer and protein content was measured using a Bradford assay (Bio-Rad, Hercules, CA). Protein extracts (20–30 μg) were resolved by SDS-PAGE (6.0 or 7.5% wt/vol) and transferred electrophoretically to PVDF membranes (Millipore). After blocking with non-dry fat milk (5% wt/vol), membranes were probed with primary antibodies. The following antibodies were used: calpain-1 domain IV (Abcam, catalog No: ab39170), calpain-2 (Abcam, catalog No: ab39165), calpain-4 (Triple Point Biologics, catalog No: RP3 Calpain-4) calpain-10 (Triple Point Biologics, catalog No: RP1 Calpain-10), calpastatin (Cell Signaling, catalog No. 4146), NF-kB phospho P-65 (Ser 536) (Cell Signaling, catalog No:3033 P), total P-65 (Cell Signaling, catalog No:8242 P), IKBα (Cell Signaling, catalog No:4814 P) and β-actin (Sigma-Aldrich, catalog No: A5441). Membranes were incubated with appropriate HRP-labeled secondary antibodies. Immune complexes were visualized by chemiluminescence (Pierce, Rockford, IL) and quantified using a Kodak Imager.

### Statistical Analyses

Data are represented as mean ± SEM. Statistical analyses were performed using SigmaPlot 12.0 (SYSTAT Software Inc., San Jose, CA, USA). Student′s *t* test or Mann-Whitney Rank Sum test was performed as appropriate for two-group comparisons. One or Two way ANOVA with Holm-Sidak post hoc analysis were performed for multiple-group and multiple-manipulation analysis. Values of *P* < 0.05 were considered to be statistically significant.

### Data Availability

The datasets generated during and/or analyzed during the current study are available from the corresponding author upon reasonable request.

## Electronic supplementary material

Supplementary Information

## References

[1] Olefsky, J. M. & Glass, C. K. Macrophages, inflammation, and insulin resistance. *Annu Rev Physiol*, 219–246 (2010). 10.1146/annurev-physiol-021909-135846 20148674

[2] Goll DE, Thompson VF, Li H, Wei W, Cong J (2003). The calpain system. Physiol Rev.

[3] Sato K (2011). Calpastatin, an endogenous calpain-inhibitor protein, regulates the cleavage of the Cdk5 activator p35 to p25. Journal of neurochemistry.

[4] Smolock AR, Mishra G, Eguchi K, Eguchi S, Scalia R (2011). Protein kinase C upregulates intercellular adhesion molecule-1 and leukocyte-endothelium interactions in hyperglycemia via activation of endothelial expressed calpain. Arterioscler Thromb Vasc Biol.

[5] Bate N (2012). Talin contains a C-terminal calpain2 cleavage site important in focal adhesion dynamics. PLoS One.

[6] Kobeissy, F. H. *et al*. Degradation of betaII-Spectrin Protein by Calpain-2 and Caspase-3 Under Neurotoxic and Traumatic Brain Injury Conditions. *Molecular neurobiology* (2014). 10.1007/s12035-014-8898-z PMC4383741 25270371

[7] Hirai S, Kawasaki H, Yaniv M, Suzuki K (1991). Degradation of transcription factors, c-Jun and c-Fos, by calpain. FEBS Lett.

[8] Carillo S (1994). Differential sensitivity of FOS and JUN family members to calpains. Oncogene.

[9] Fenouille N (2012). Calpain 2-dependent IkappaBalpha degradation mediates CPT-11 secondary resistance in colorectal cancer xenografts. The Journal of pathology.

[10] Kerbiriou M, Teng L, Benz N, Trouve P, Ferec C (2009). The calpain, caspase 12, caspase 3 cascade leading to apoptosis is altered in F508del-CFTR expressing cells. PLoS One.

[11] Wolf BB (1999). Calpain functions in a caspase-independent manner to promote apoptosis-like events during platelet activation. Blood.

[12] Ma J (2012). Deficiency of Capn4 gene inhibits nuclear factor-kappaB (NF-kappaB) protein signaling/inflammation and reduces remodeling after myocardial infarction. J Biol Chem.

[13] Wendt A, Thompson VF, Goll DE (2004). Interaction of calpastatin with calpain: a review. Biol Chem.

[14] Huang Z, Hoffmann FW, Norton RL, Hashimoto AC, Hoffmann PR (2011). Selenoprotein K is a novel target of m-calpain, and cleavage is regulated by Toll-like receptor-induced calpastatin in macrophages. J Biol Chem.

[15] Scalia R (2011). A novel role for calpain in the endothelial dysfunction induced by activation of angiotensin II type 1 receptor signaling. Circ Res.

[16] Letavernier E (2008). Targeting the calpain/calpastatin system as a new strategy to prevent cardiovascular remodeling in angiotensin II-induced hypertension. Circ Res.

[17] Huang Z (2013). Calpastatin prevents NF-kappaB-mediated hyperactivation of macrophages and attenuates colitis. Journal of immunology.

[18] Subramanian V (2012). Calpain Inhibition Attenuates Angiotensin II-induced Abdominal Aortic Aneurysms and Atherosclerosis in Low-density Lipoprotein Receptor-deficient Mice. J Cardiovasc Pharmacol.

[19] Subramanian V (2013). Calpain-2 compensation promotes angiotensin II-induced ascending and abdominal aortic aneurysms in calpain-1 deficient mice. PLoS One.

[20] del Bosque-Plata L (2004). Association of the calpain-10 gene with type 2 diabetes mellitus in a Mexican population. Mol Genet Metab.

[21] Cheverud JM (2010). Calpain-10 is a component of the obesity-related quantitative trait locus Adip1. J Lipid Res.

[22] Momeni HR (2011). Role of calpain in apoptosis. Cell J.

[23] Sharma AK, Rohrer B (2004). Calcium-induced calpain mediates apoptosis via caspase-3 in a mouse photoreceptor cell line. J Biol Chem.

[24] Strissel KJ (2007). Adipocyte death, adipose tissue remodeling, and obesity complications. Diabetes.

[25] Cinti S (2005). Adipocyte death defines macrophage localization and function in adipose tissue of obese mice and humans. J Lipid Res.

[26] Alkhouri N (2010). Adipocyte apoptosis, a link between obesity, insulin resistance, and hepatic steatosis. J Biol Chem.

[27] Park SH (2016). IKKbeta Is Essential for Adipocyte Survival and Adaptive Adipose Remodeling in Obesity. Diabetes.

[28] Aubin K (2015). Characterization of *In Vitro* Engineered Human Adipose Tissues: Relevant Adipokine Secretion and Impact of TNF-alpha. PLoS One.

[29] Howatt DA (2016). Leukocyte Calpain Deficiency Reduces Angiotensin II-induced Inflammation and Atherosclerosis but not Abdominal Aortic Aneurysms in Mice. Arterioscler Thromb Vasc Biol.

[30] Sui Y (2014). IKKbeta links vascular inflammation to obesity and atherosclerosis. The Journal of experimental medicine.

[31] Baker RG, Hayden MS, Ghosh S (2011). NF-kappaB, inflammation, and metabolic disease. Cell Metab.

[32] Perkins ND (2007). Integrating cell-signalling pathways with NF-kappaB and IKK function. Nature reviews. Molecular cell biology.

[33] Shumway SD, Maki M, Miyamoto S (1999). The PEST domain of IkappaBalpha is necessary and sufficient for *in vitro* degradation by mu-calpain. J Biol Chem.

[34] Chen F (2000). Impairment of NF-kappaB activation and modulation of gene expression by calpastatin. American journal of physiology. Cell physiology.

[35] Divoux A (2010). Fibrosis in human adipose tissue: composition, distribution, and link with lipid metabolism and fat mass loss. Diabetes.

[36] Marcelin G (2017). A PDGFRalpha-Mediated Switch toward CD9high Adipocyte Progenitors Controls Obesity-Induced Adipose Tissue Fibrosis. Cell Metab.

[37] Gomez-Valades AG (2008). Pck1 gene silencing in the liver improves glycemia control, insulin sensitivity, and dyslipidemia in db/db mice. Diabetes.

[38] Franckhauser S, Munoz S, Elias I, Ferre T, Bosch F (2006). Adipose overexpression of phosphoenolpyruvate carboxykinase leads to high susceptibility to diet-induced insulin resistance and obesity. Diabetes.

[39] Miyazaki T (2011). m-Calpain Induction in Vascular Endothelial Cells on Human and Mouse Atheromas and Its Roles in VE-Cadherin Disorganization and Atherosclerosis. Circulation.

[40] Pereyra AS (2017). BDA-410 Treatment Reduces Body Weight and Fat Content by Enhancing Lipolysis in Sedentary Senescent Mice. J Gerontol A Biol Sci Med Sci.

[41] Hanna RA, Campbell RL, Davies PL (2008). Calcium-bound structure of calpain and its mechanism of inhibition by calpastatin. Nature.

[42] Suzuki K, Hata S, Kawabata Y, Sorimachi H (2004). Structure, activation, and biology of calpain. Diabetes.

[43] Kwon H (2014). Adipocyte-specific IKKbeta signaling suppresses adipose tissue inflammation through an IL-13-dependent paracrine feedback pathway. Cell Rep.

[44] Donadelli R (2000). Protein traffic activates NF-kB gene signaling and promotes MCP-1-dependent interstitial inflammation. American journal of kidney diseases: the official journal of the National Kidney Foundation.

[45] Rego D (2011). IL-6 production is positively regulated by two distinct Src homology domain 2-containing tyrosine phosphatase-1 (SHP-1)-dependent CCAAT/enhancer-binding protein beta and NF-kappaB pathways and an SHP-1-independent NF-kappaB pathway in lipopolysaccharide-stimulated bone marrow-derived macrophages. Journal of immunology.

[46] Khan T (2009). Metabolic dysregulation and adipose tissue fibrosis: role of collagen VI. Mol Cell Biol.

[47] Itoh M (2011). Melanocortin 4 receptor-deficient mice as a novel mouse model of nonalcoholic steatohepatitis. Am J Pathol.

[48] Tanaka M (2014). Macrophage-inducible C-type lectin underlies obesity-induced adipose tissue fibrosis. Nat Commun.

[49] Peltier J (2006). Calpain activation and secretion promote glomerular injury in experimental glomerulonephritis: evidence from calpastatin-transgenic mice. J Am Soc Nephrol.

[50] Daugherty A, Rateri DL, Lu H, Inagami T, Cassis LA (2004). Hypercholesterolemia stimulates angiotensin peptide synthesis and contributes to atherosclerosis through the AT1A receptor. Circulation.

[51] Lu H, Rateri DL (2007). Immunostaining in mouse atherosclerosis. Methods Mol Biol.

[52] Putnam K (2012). Deficiency of angiotensin type 1a receptors in adipocytes reduces differentiation and promotes hypertrophy of adipocytes in lean mice. Endocrinology.

[53] Fan H (2011). Macrophage migration inhibitory factor and CD74 regulate macrophage chemotactic responses via MAPK and Rho GTPase. Journal of immunology.

[54] Cassis LA, Rateri DL, Lu H, Daugherty A (2007). Bone marrow transplantation reveals that recipient AT1a receptors are required to initiate angiotensin II-induced atherosclerosis and aneurysms. Arterioscler Thromb Vasc Biol.

[55] Gupta RK (2010). Transcriptional control of preadipocyte determination by Zfp423. Nature.

