# Supplementary material for: Calpain Inhibition Attenuates Adipose Tissue Inflammation and Fibrosis in Diet-induced Obese Mice
Source: Sci Rep. 2017 Oct 31;7:14398. doi: 10.1038/s41598-017-14719-9 (PMC5663911; doi:10.1038/s41598-017-14719-9)
Supplement: Supplementary file 1 — Supplementary Information [file 41598_2017_14719_MOESM1_ESM.pdf]

Supplementary Information

Calpain Inhibition Attenuates Adipose Tissue Inflammation and Fibrosis in Diet-induced Obese Mice

Latha Muniappan[1^], Aida Javidan[1], Weihua Jang[1], Shayan Mohammadmoradi[1], Jessica J. Moorleghen[1], Wendy S. Katz[2], Anju Balakrishnan[1], Deborah A. Howatt[1], Venkateswaran Subramanian[1,2]*.

[1]Saha Cardiovascular Research Center, University of Kentucky, Lexington, KY, USA.
[2]Department of Physiology, University of Kentucky, Lexington, KY, USA.

^ Deceased

*** Address for Correspondence:**

Venkateswaran Subramanian
Saha Cardiovascular Research Center
Department of Physiology
BBSRB - Room 247
University of Kentucky
Lexington, KY 40536-0509
E-mail: venkat.subramanian@uky.edu

Online Supplement

Number of Figures	:	8
Number or Tables	:	2

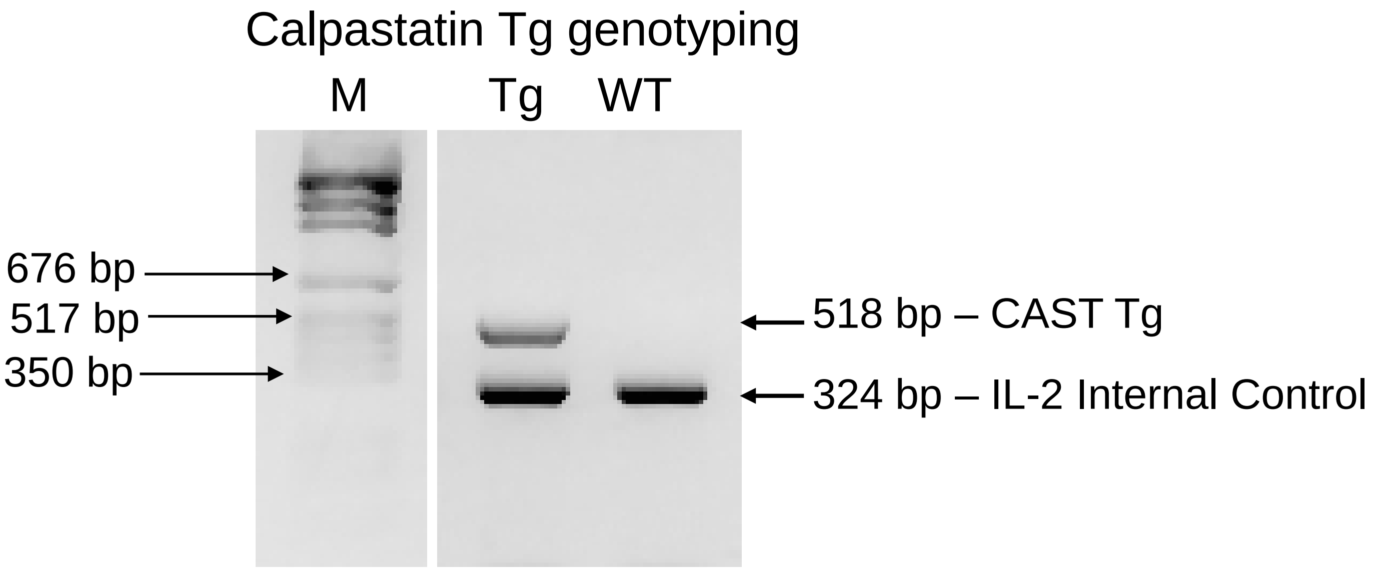

Supplementary Figure I. Genotyping of experimental mice for the CAST transgene (Tg) by PCR.
A. Genomic DNA from tail was isolated and screened by PCR for the CAST-Tg allele. IL-2 was used as an internal control. PCR on tail DNA yielded amplicons of 518 and 324 bp for CAST-Tg and IL-2 alleles, respectively. (M= Molecular weight ladder)

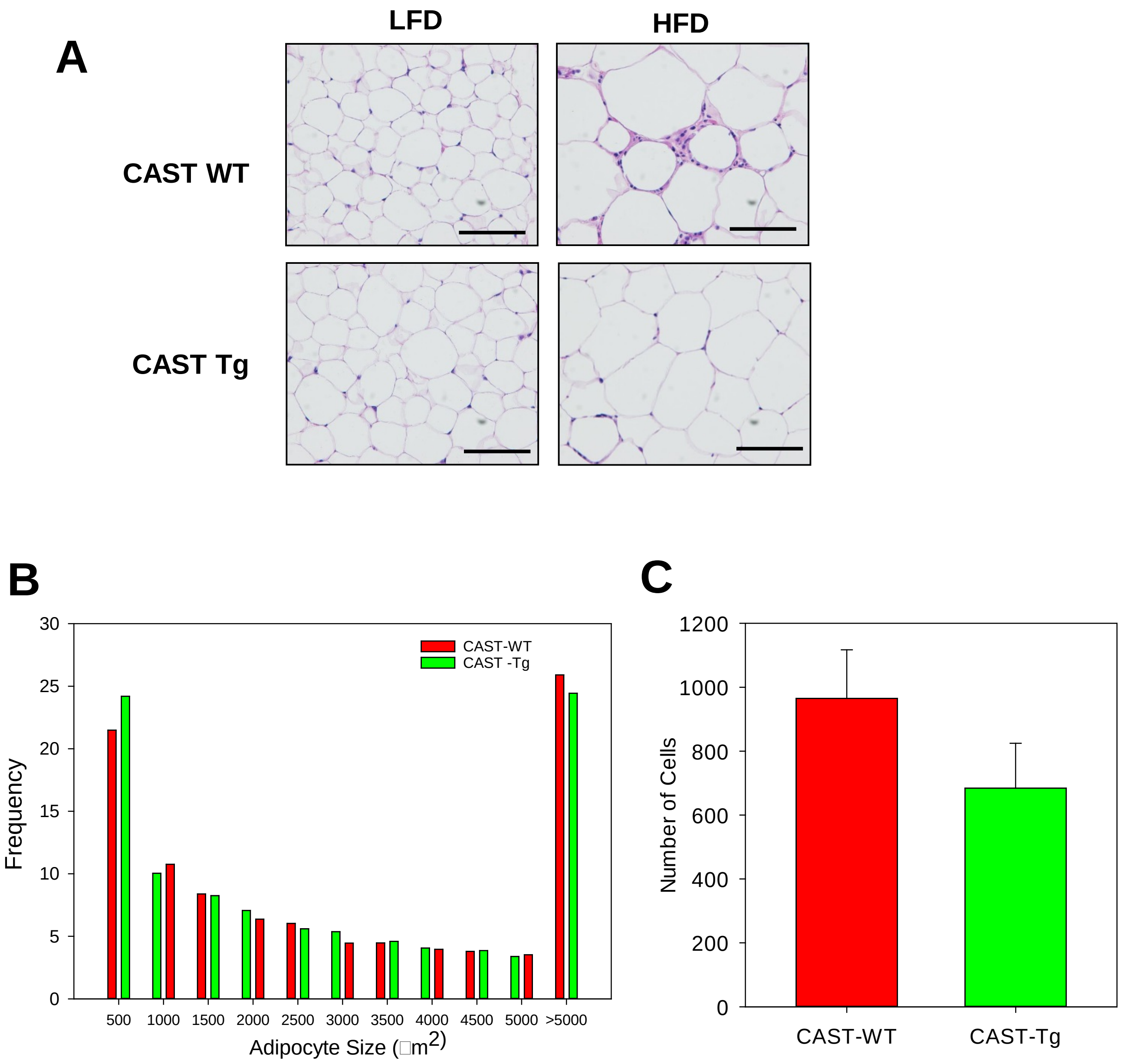

Supplementary Figure II. CAST overexpression had no effect on obesity-induced adipocyte expansion. A. Representative hematoxylin and eosin staining of EpiWAT cross-sections from LFD and HFD fed CAST WT and Tg mice. **B.** Histogram of adipocyte diameters in EpiWAT from HFD fed CAST WT and Tg mice. **C.** Quantification of adipocyte number per measurement frame (n=5-6 mice). Values are represented as mean ± SEM.

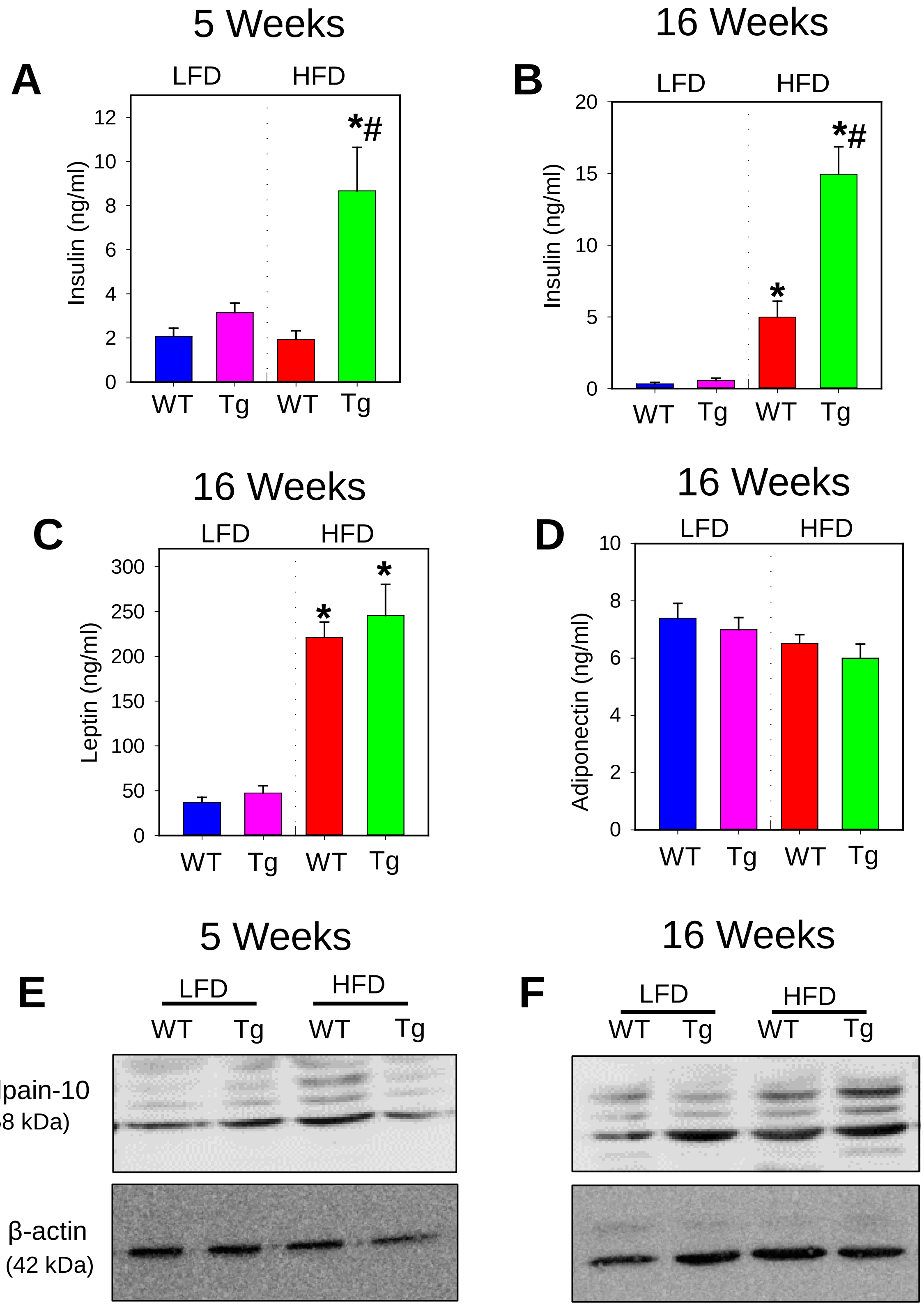

Supplementary Figure III. CAST overexpression increased plasma insulin levels upon HFD feeding. Plasma Insulin **(A,B)**, leptin (**C**) and adiponectin (**D**) were measured by ELISA (n=12-14). Values are represented as mean ± SEM. * denotes $P<0.05$ HFD vs LFD; #$P<0.05$ CAST WT vs CAST Tg (Two-way ANOVA with Holm-Sidak post hoc analysis). **E, F.** Calpain-10 protein was detected by Western blotting of EpiWAT extracts from WT and CAST Tg mice fed a LFD or HFD diet for 5 weeks or 16 weeks (n=4).

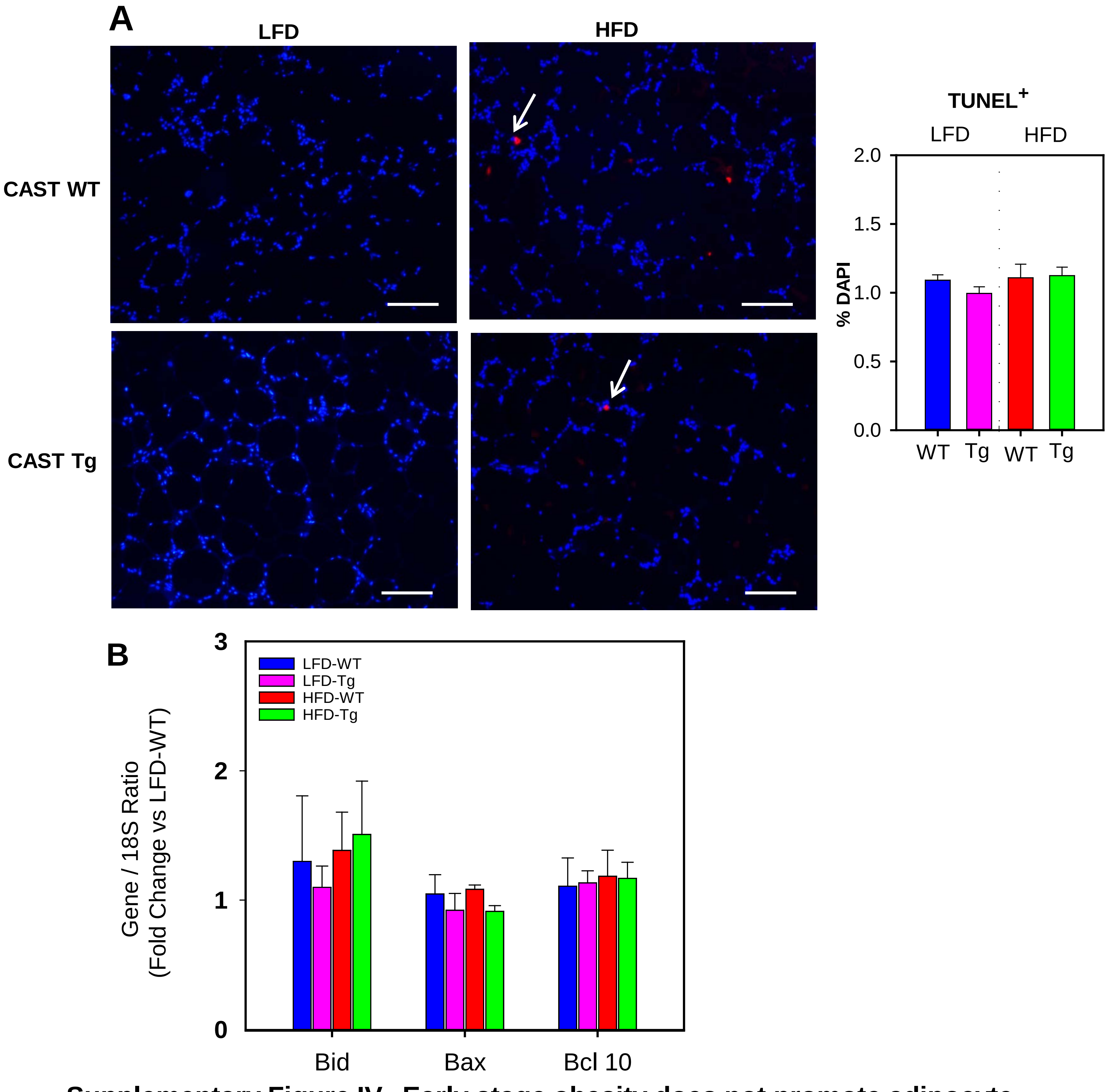

Supplementary Figure IV. Early stage obesity does not promote adipocyte cell death. A. Representative TUNEL staining of EpiWAT cross-sections from 5 weeks LFD and HFD fed CAST WT and Tg mice. The nuclei were stained with DAPI (blue) and the TUNEL-positive cells (red) were indicated by arrows. Under fluorescent microscopy, TUNEL-positive cells were counted from 10 fields at the power of 100x magnification (n=5). **B.** mRNA abundance of Bid, Bax and Bcl 10 genes in EpiWAT from LFD and HFD fed CAST WT and Tg mice were analyzed by qPCR (n=4-6). Values are represented as mean ± SEM (Two-way ANOVA with Holm-Sidak post hoc analysis).

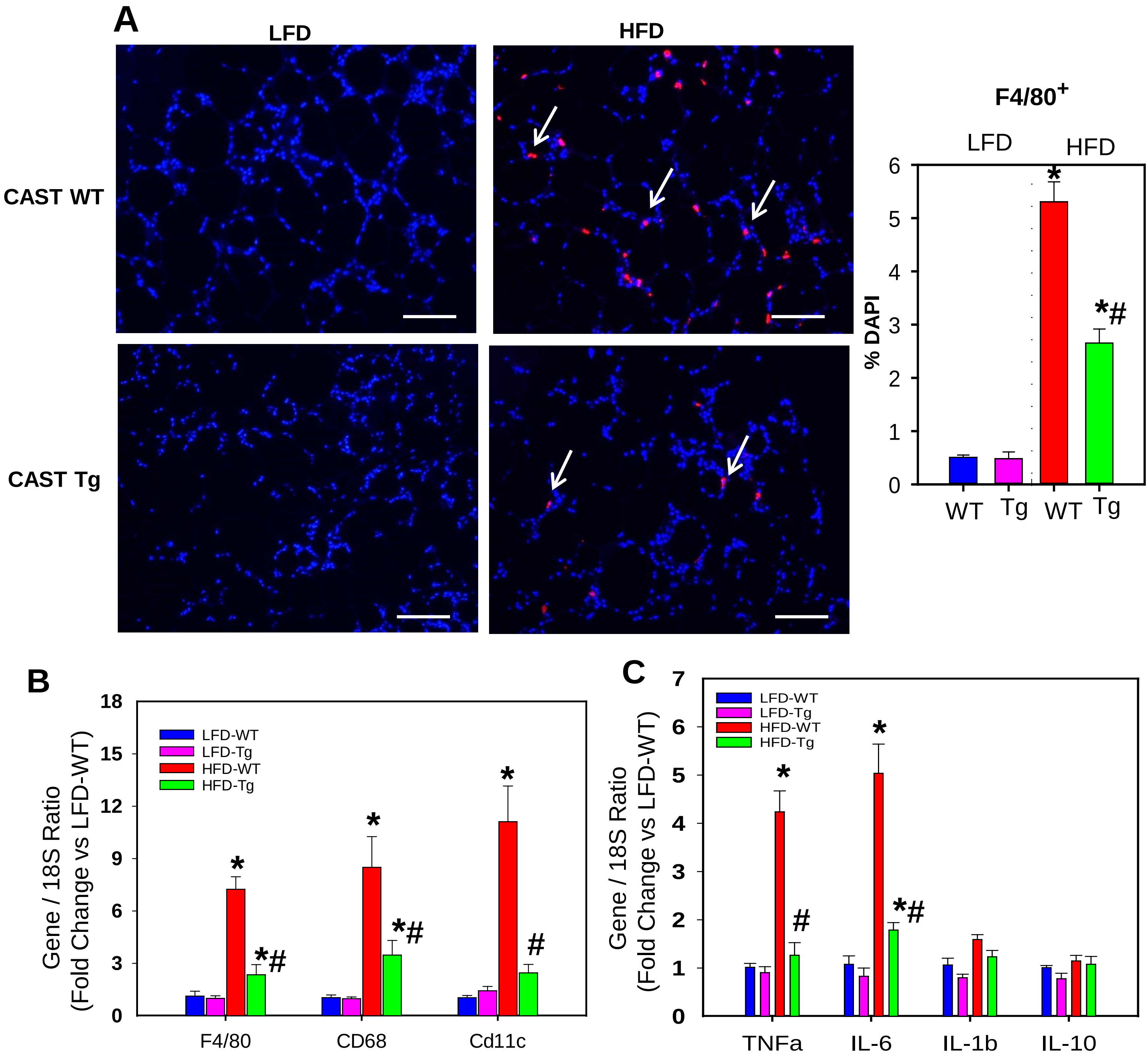

Supplementary Figure V. CAST overexpression significantly reduced macrophage accumulation and inflammatory genes during the early stage of obesity. A. Representative immunofluorescent staining of F4/80 in EpiWAT cross-sections from 5 weeks LFD and HFD fed CAST WT and Tg mice. The nuclei were stained with DAPI (blue) and the F4/80-positive cells (red) were indicated by arrows. Using fluorescent microscopy, F4/80-positive cells were counted from 10 fields at the power of 100x magnification (n=5). **B.** mRNA abundance of F4/80, CD68, and CD11c genes in EpiWAT from LFD and HFD fed CAST WT and Tg mice were analyzed by qPCR (n=5). **C.** mRNA abundance of TNFα, IL-6, IL-1β, and IL-10 genes in EpiWAT from LFD and HFD fed CAST WT and Tg mice (n=5). * denotes *P*<0.05 when comparing LFD vs HFD (Two-way ANOVA with Holm-Sidak post hoc analysis).

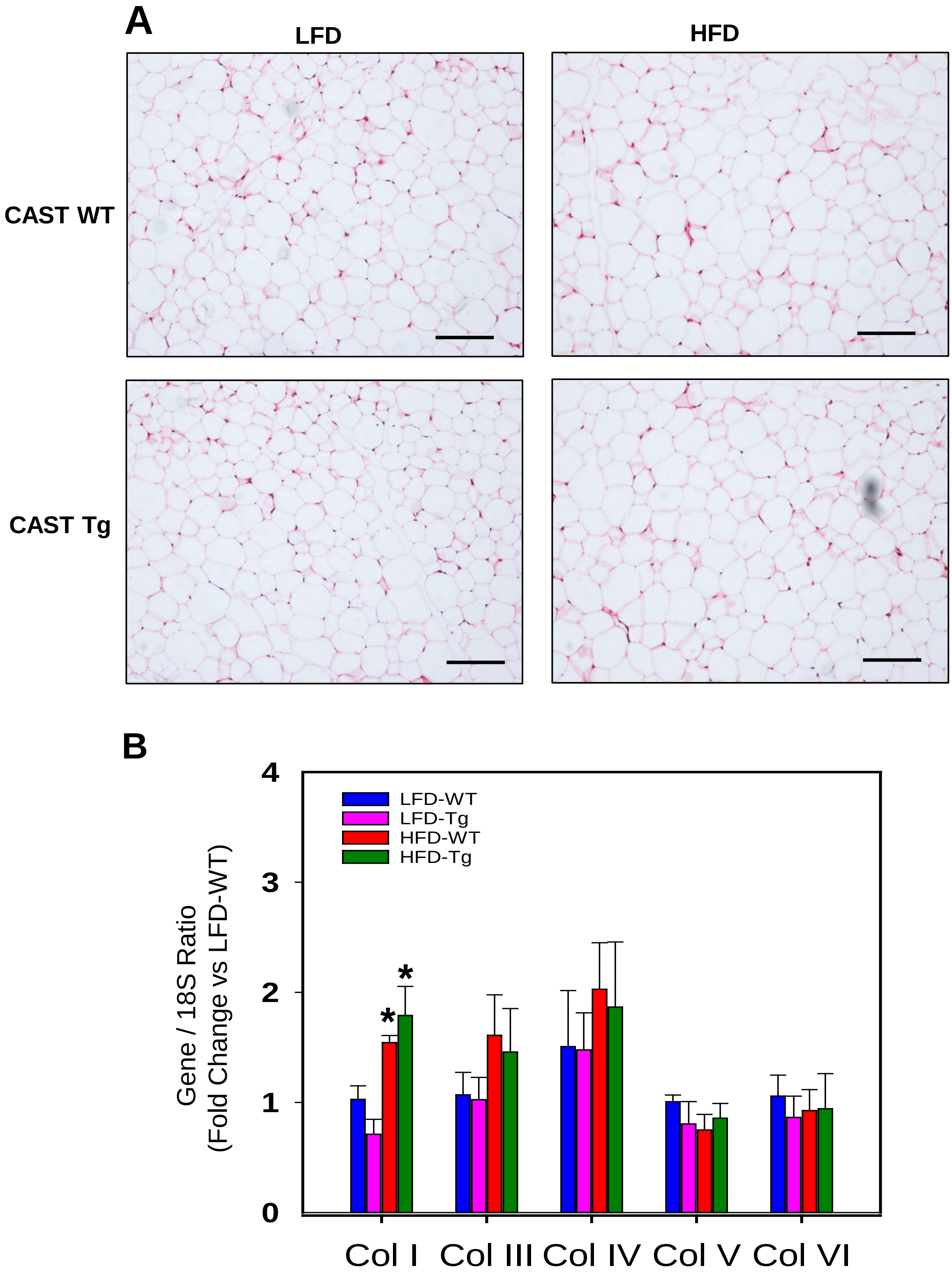

Supplementary Figure VI. Early stage obesity showed no change in interstitial fibrosis in adipose tissue. A. Representative Sirius red staining of EpiWAT cross-sections from 5 weeks LFD and HFD fed CAST WT and Tg mice. **B**. mRNA abundance of Col I, Col III, Col IV, Col V and Col VI genes in EpiWAT from LFD and HFD fed CAST WT and Tg mice were analyzed by qPCR (n=5). Values are represented as mean ± SEM. * denotes $P<0.05$ when comparing LFD vs HFD (Two-way ANOVA with Holm-Sidak post hoc analysis).

A

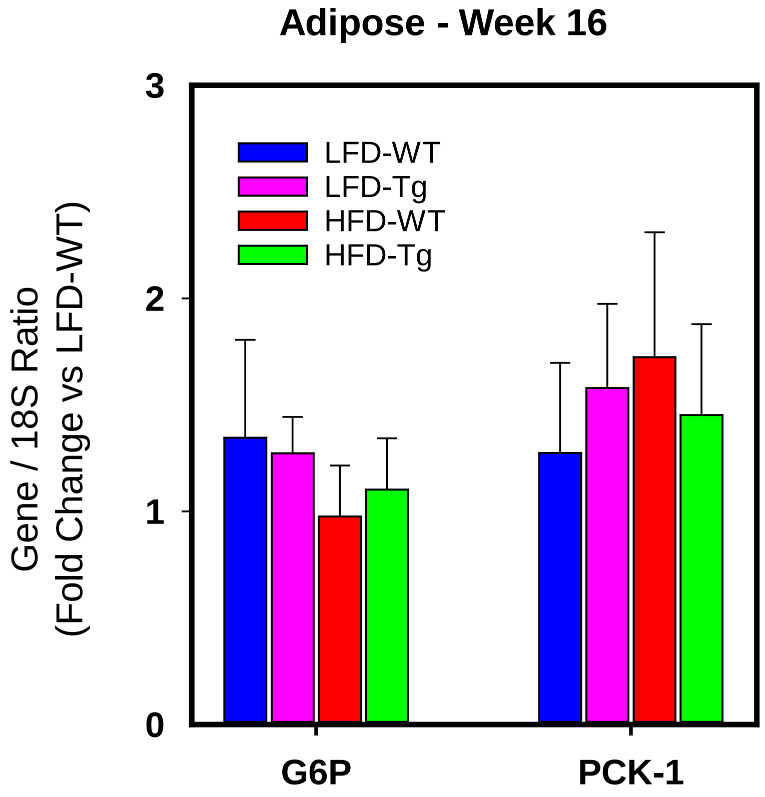

B

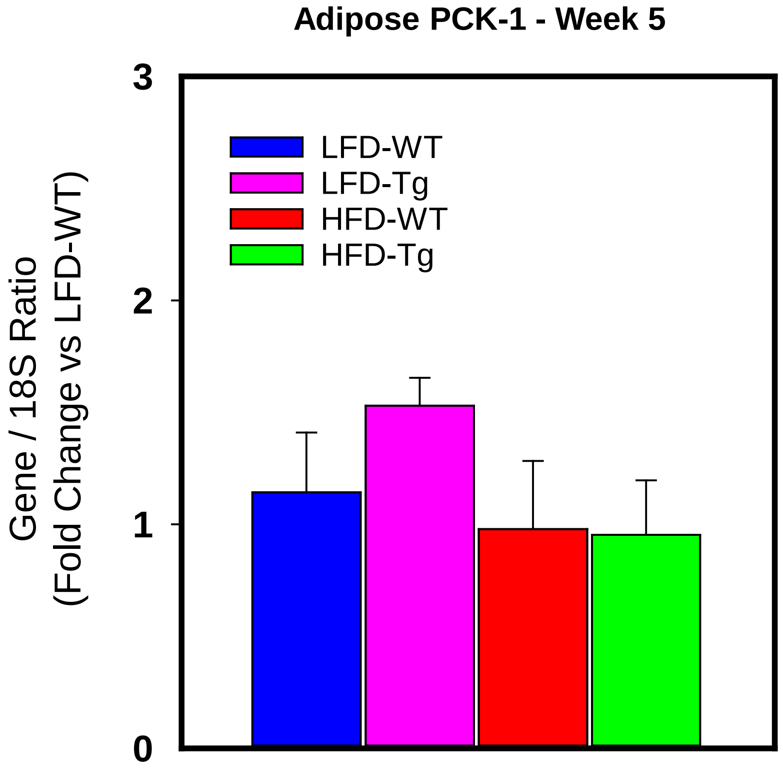

Supplementary Figure VII. CAST overexpression had no effect on adipose tissue PCK-1 mRNA abundance. mRNA abundance of G6P and PCK-1 in adipose tissue from 16 week (**A**) and PCK-1 in adipose tissue from 5 week (**B**) LFD and HFD fed CAST WT and Tg mice were analyzed by qPCR (n=5). Values are represented as mean ± SEM. (Two-way ANOVA with Holm-Sidak post hoc analysis).

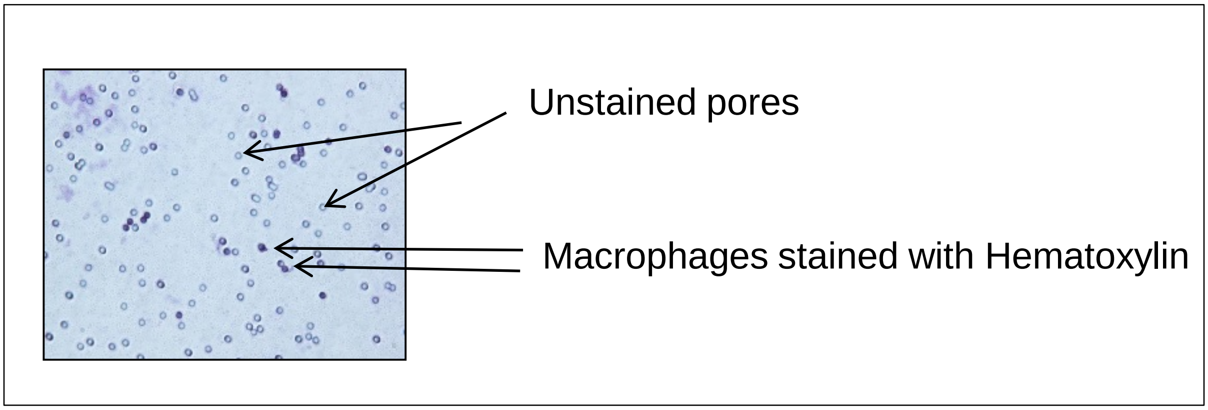

Supplementary Figure VIII. An example of the transwell membrane migrated macrophages stained with hematoxylin.

Supplementary Table I. Effects of calpastatin overexpression on food and water intake in LFD and HFD fed mice

Groups	CAST WT		CAST Tg	
**Diet**	**LFD**	**HFD**	**LFD**	**HFD**
N	10	12	10	13
Food Intake (g/day)	4.3 ± 0.18	3.5 ± 0.08	4.1 ± 0.13	3.1 ± 0.15
Water Intake (ml/day)	4.2 ± 0.88	3.0 ± 0.49	3.9 ± 0.72	3.2 ± 0.53

Values are represented as means ± SEMs.

Supplementary Table II. Primers used for real-time PCR

Gene	Primers	Product size (bp)
Bid	5'-CTGCCTGTGCAAGCTTACTG-3'<br>5'-GTCTGGCAATGTTGTGGATG-3'	142
Bax	5'-TGTTTGCTGATGGCAACTTC-3'<br>5'-GATCAGCTCGGGCACTTTAG-3'	104
Bcl 10	5'-GCAGGGAGAAAACACAGAGC-3'<br>5'-CGGAATTGCACCTAGAGAGG-3'	156
Herpud 1	5'-CCTGGCTTCTCTGGCTACAC-3'<br>5'-GTCGGGACAAAAGTTCCTGA-3'	104
F4/80	5'-CTGTAACCGGATGGCAAACT-3'<br>5'-ATGGCCAAGGCAAGACATAC-3'	123
CD68	5'-CCAATTCAGGGTGGAAGAAA-3'<br>5'-ATGGGTACCGTCACAACCTC-3'	114
CD11c	5'-CAAAATCTCCAACCCATGCT-3'<br>5'-TCTGGGAAGCCAAATACGAC-3'	128
CD206	5'-CAAGGAAGGTTGGCATTTGT-3'<br>5'-CCTTTCAGTCCTTTGCAAGC-3'	111
MCP-1	5'-CAGCCAGATGCAGTTAACGC -3'<br>5'-TCTGGACCCATTCCTTCTTG-3'	175
CCR2	5'-ACACCCTGTTTCGCTGTAGG-3'<br>5'-GATTCCTGGAAGGTGGTCAA-3'	133
TNFα	5'-CCCACTCTGACCCCTTTACTC-3'<br>5'-TCACTGTCCCAGCATCTTGT-3'	114
IL-6	5'-GGGAAATCGTGGAAATGAGAAA-3'<br>5'-AAGTGCATCATCGTTGTTCATACA-3'	167
IL-1β	5'-TCGTGCTGTCGGACCCATAT-3'<br>5'-GTCGTTGCTTGGTTCTCCTTGT-3'	110
IL-10	5'-CCAAGCCTTATCGGAAATGA-3'<br>5'-TCTCACCCAGGGAATTCAAA-3'	190
Col I	5'-TGGCAACAAAGGAGACACTG-3'<br>5'-GGCTCCTCGTTTTCCTTCTT-3'	97
Col III	5'-ACCAAAAGGTGATGCTGGAC-3'<br>5'-GACCTCGTGCTCCAGTTAGC-3'	110
Col IV	5'-CCAAAGGATCAGTTGGAGGA-3'<br>5'-CTCTCCTTTGGCTCCCTTCT-3'	119
Col V	5'-GGTCCCTGACACACCTCAGT-3'<br>5'-TGCTCCTCAGGAACCTCTGT-3'	183

Col VI	5'-GGGACACGACACCTCTCAGT-3'<br>5'-TTGGCAGGAAATGACATTGA-3'	119
ABCA1	5'-CTGGTTTGGTGAGGAAATTCA-3'<br>5'-ACCTTCATGCCATCTCGGTA-3'	150
ABCG1	5'-GCTGGGAAGTCCACACTCAT-3’<br>5'-ATCATGGGTCCTGAAGAGT-3’	173
FAS	5'-CCAGTGTCCACCACCAAGCG-3’<br>5'-GGAGCGCAGGATAGACTCAC-3’	111
FABP	5'-CAATAGGTCTGCCCGAGGAC-3’<br>5'-AGCTTGACGACTGCCTTGAC-3’	193
G6P	5'-TGGATTCCGGTGTTTGAACG-3’<br>5'-GCAAGGTAGATCCGGGACAG-3’	84
PCK-1	5'-ATGAAAGGCCGCACCATGTA-3’<br>5'-AGGCCCAGTTGTTGACCAAA-3’	235
PPARγ	5’- AGCATCAGGCTTCCACTATG -3’<br>5’- ATCCGGCAGTTAAGATCACA-3’	111
Adiponectin	5’-GGAACTTGTGCAGGTTGGAT-3’<br>5’-CGAATGGGTACATTGGGAAC-3’	293
CEBPα	5’-TGGACAAGAACAGCAACGAG-3’<br>5’-TCACTGGTCAACTCCAGCAC-3’	127
CEBPβ	5’-CAAGCTGAGCGACGAGTACA-3’<br>5’-AGCTGCTCCACCTTCTTCTG-3’	156
PREF-1	5’-GGCAGTGCATCTGCAAGGAT-3’<br>5’-CAGGTCCACGCAAGTTCCAT-3’	104
UCP-1	5’-TGGAAAGGGACGACCCCTAA-3’<br>5’-CAGGAGTGTGGTGCAAAACC-3’	168
18S	5'-CTCTGTTCCGCCTAGTCCTG<br>5'-AATGAGCCATTCGCAGTTTC	170

Full-length blot of representative Western Blot images of Figure 1A, 1D, 1E, 1F and Figure 5B and 5E.

Figure 1

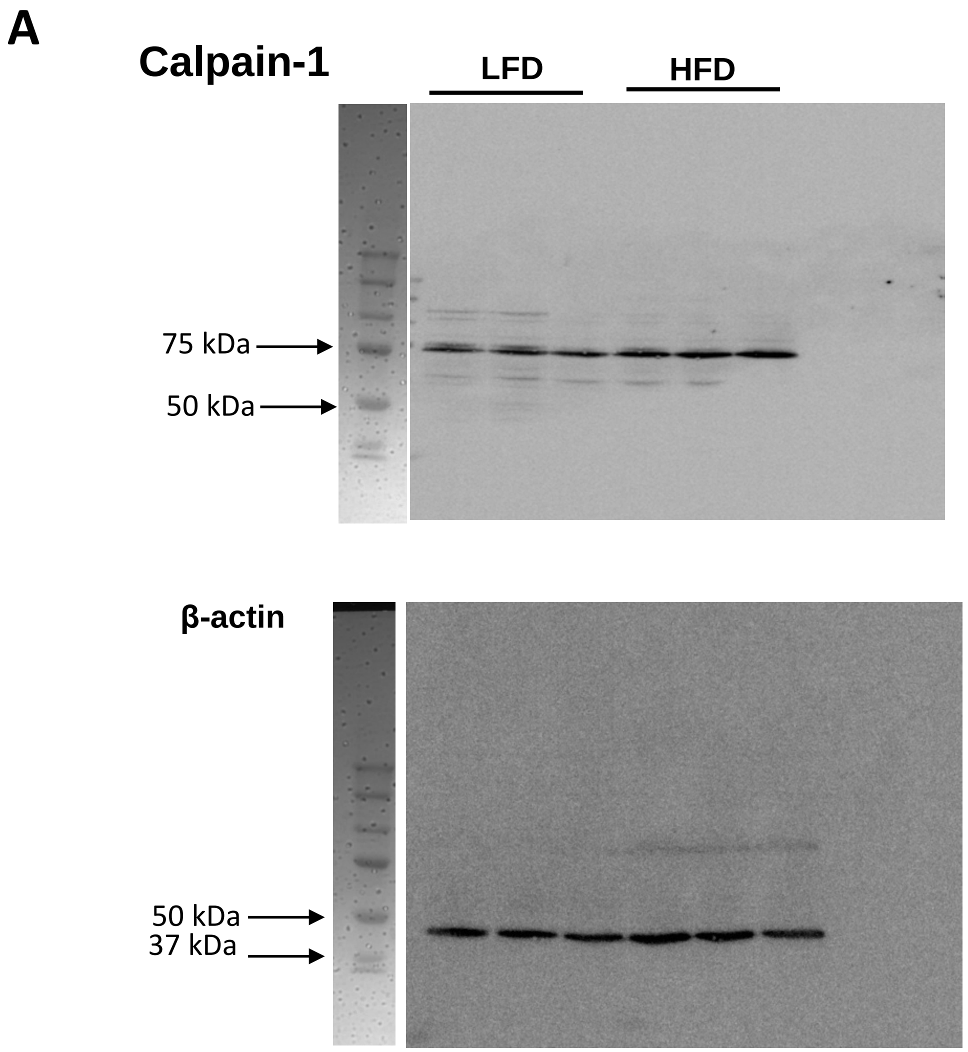

Figure 1

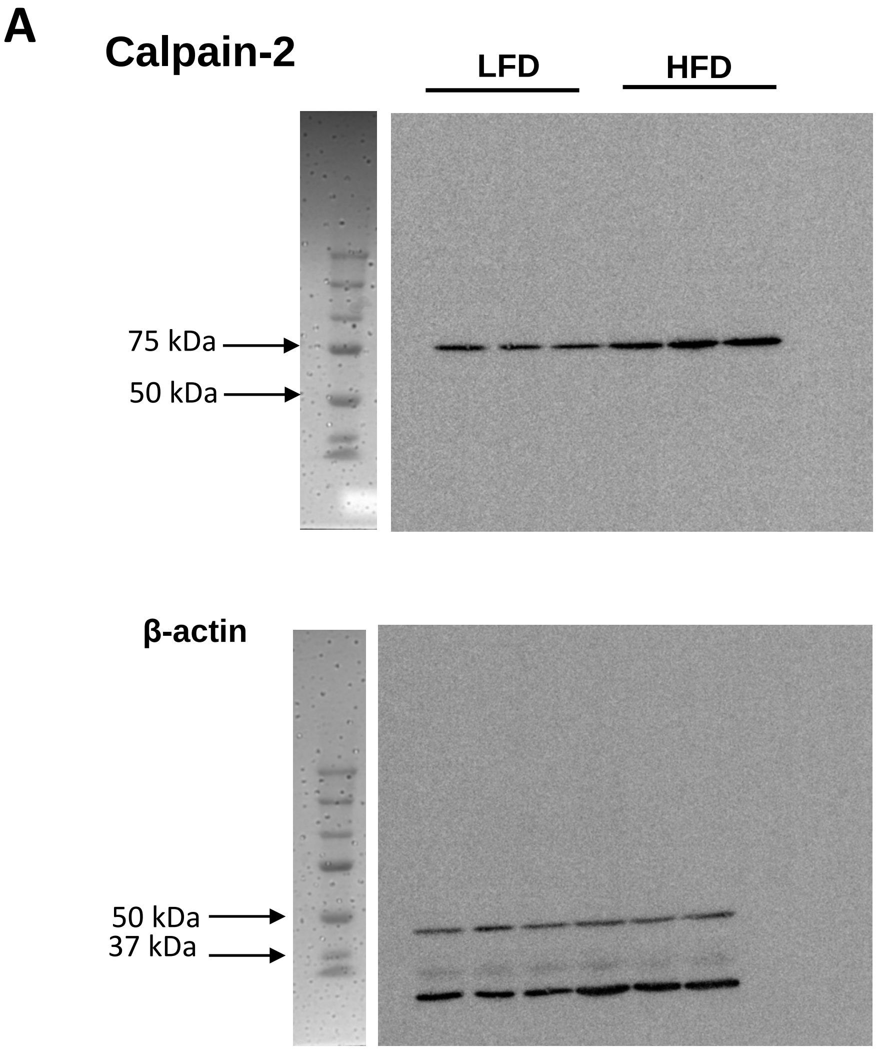

Figure 1

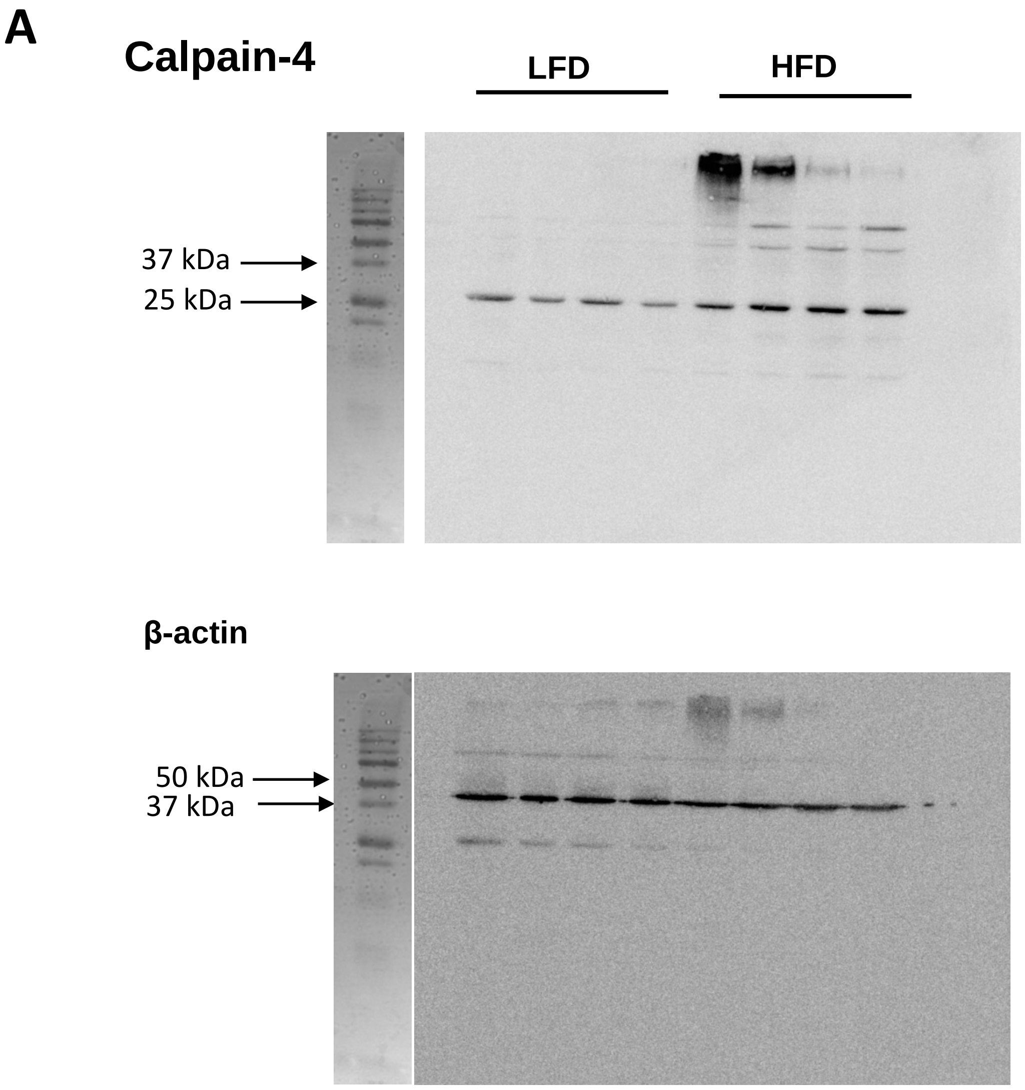

Figure 1

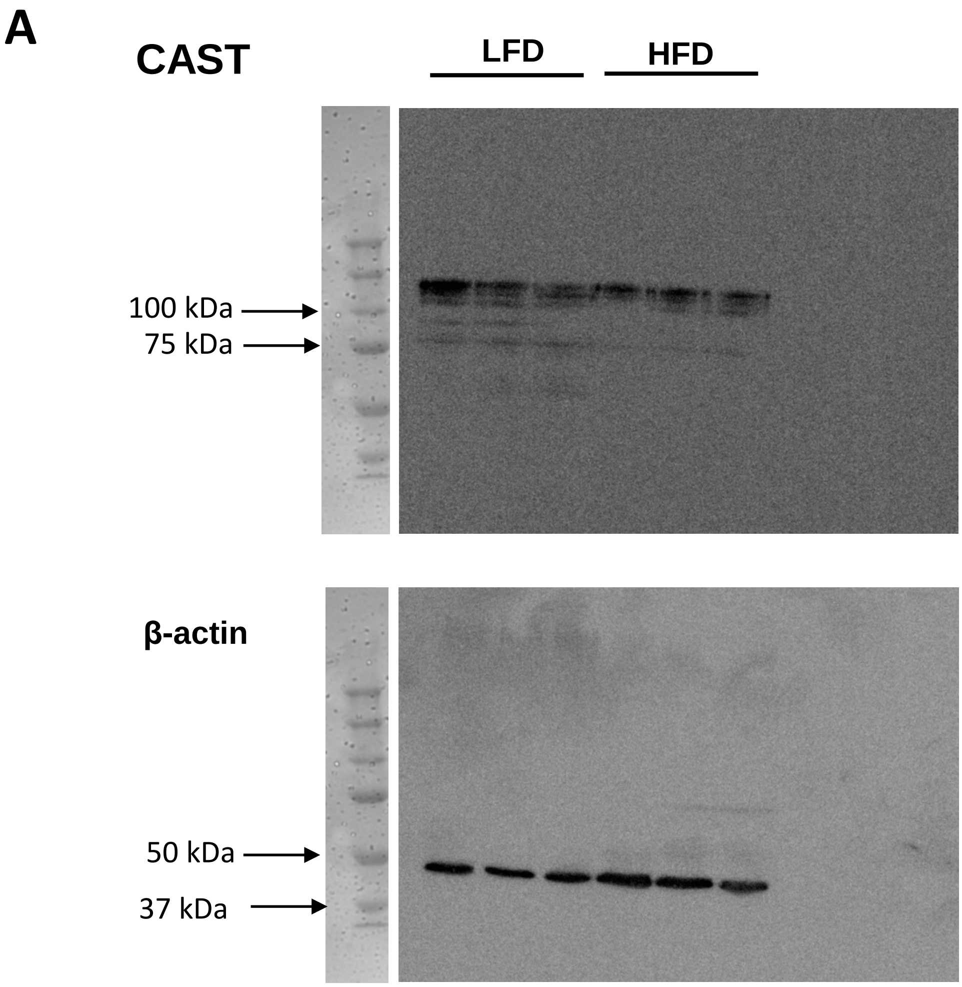

Figure 1

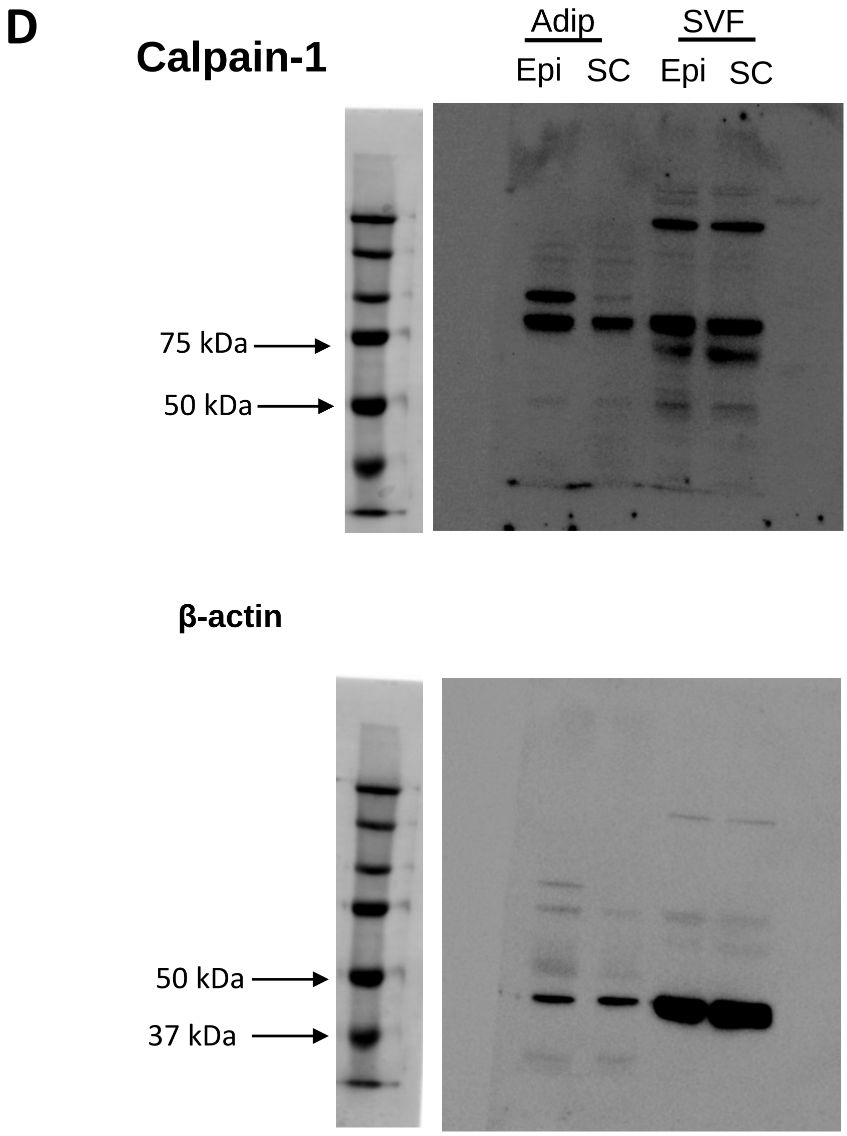
D
Calpain-1
Adip
SVF
Epi
SC
Epi
SC
75 kDa
50 kDa
β-actin
50 kDa
37 kDa

Figure 1

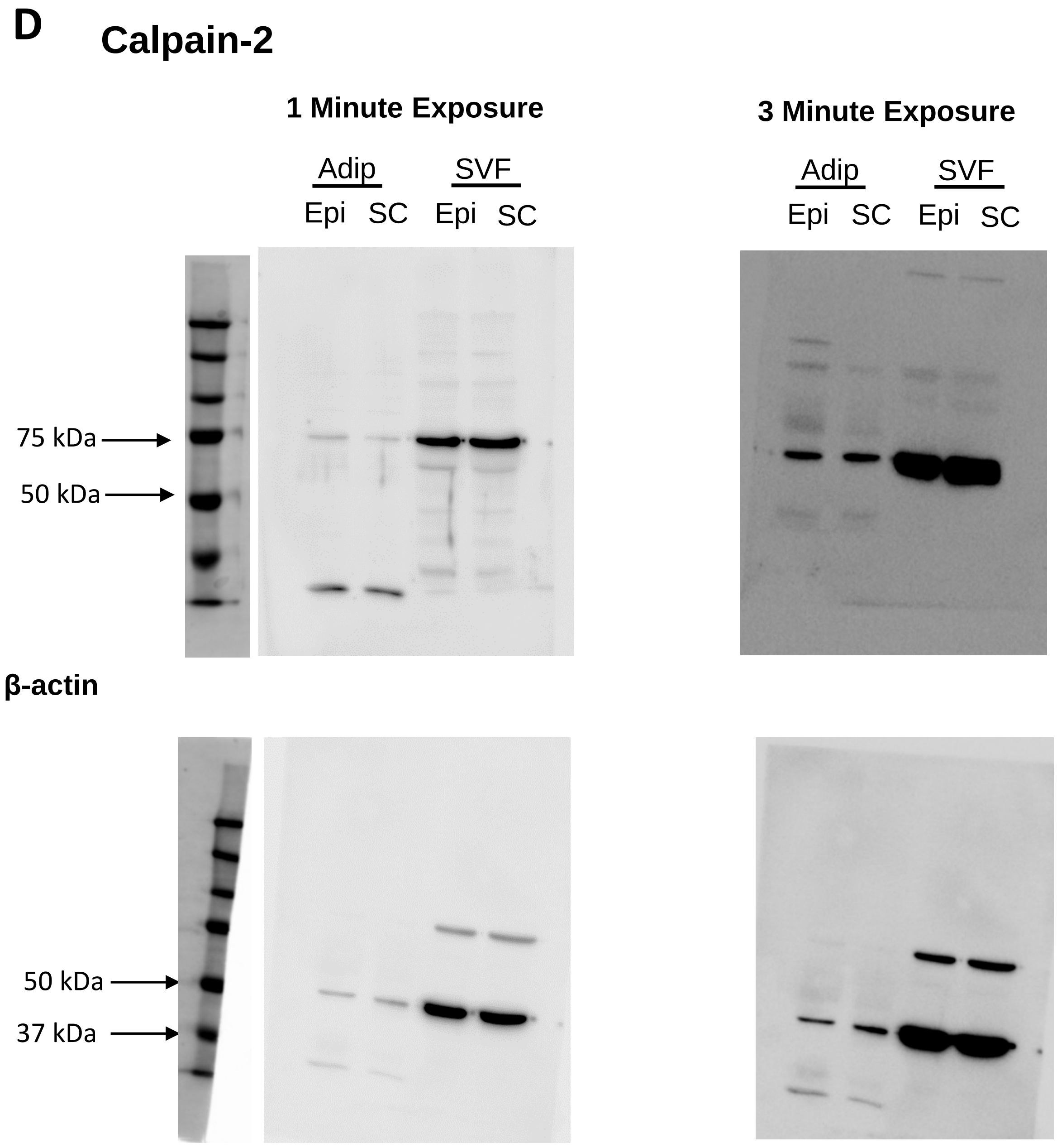
D
Calpain-2
1 Minute Exposure
3 Minute Exposure
Adip
SVF
Epi
SC
Epi
SC
Adip
SVF
Epi
SC
Epi
SC
75 kDa
50 kDa
β-actin
50 kDa
37 kDa

Figure 1

D Calpain-4

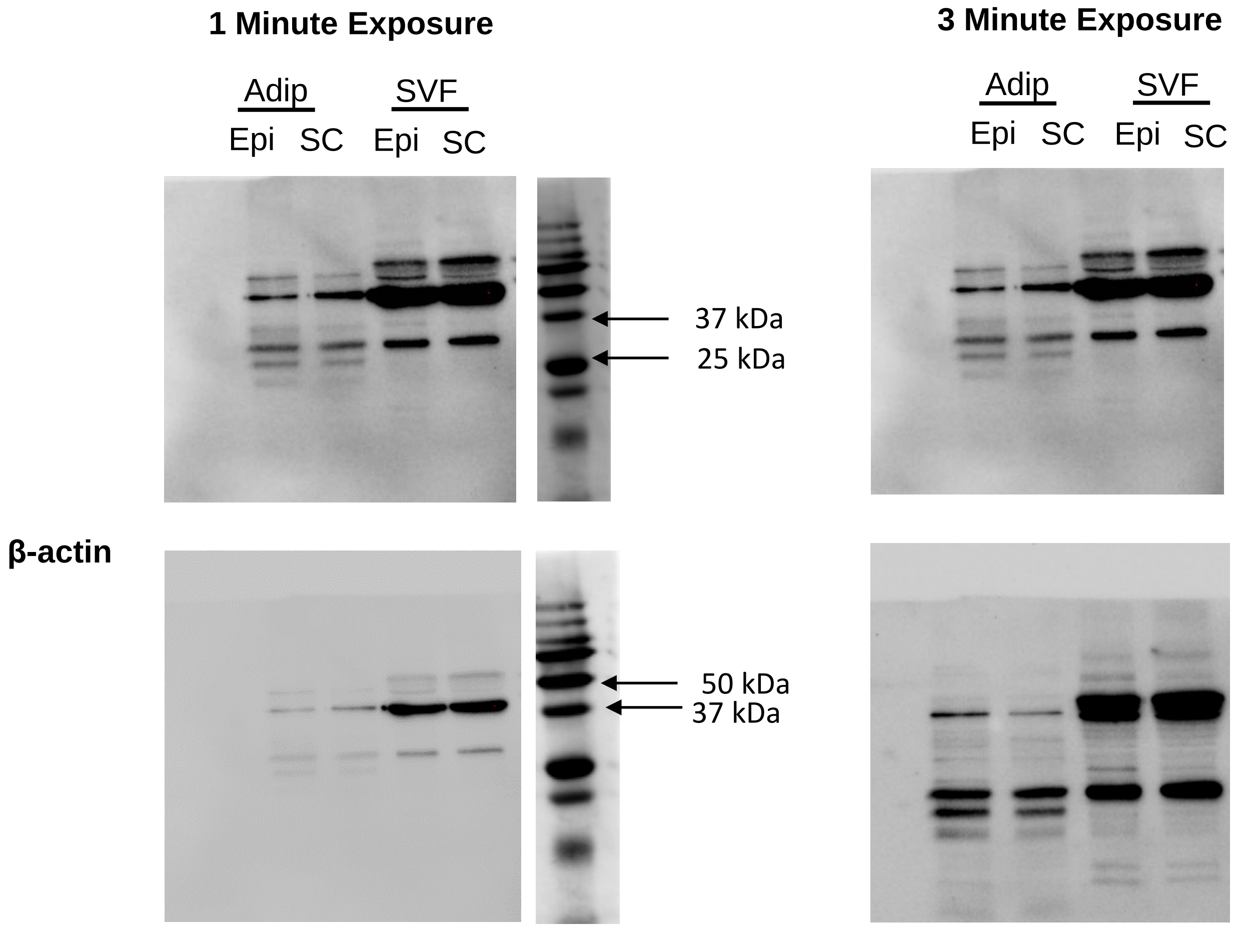

Figure 1

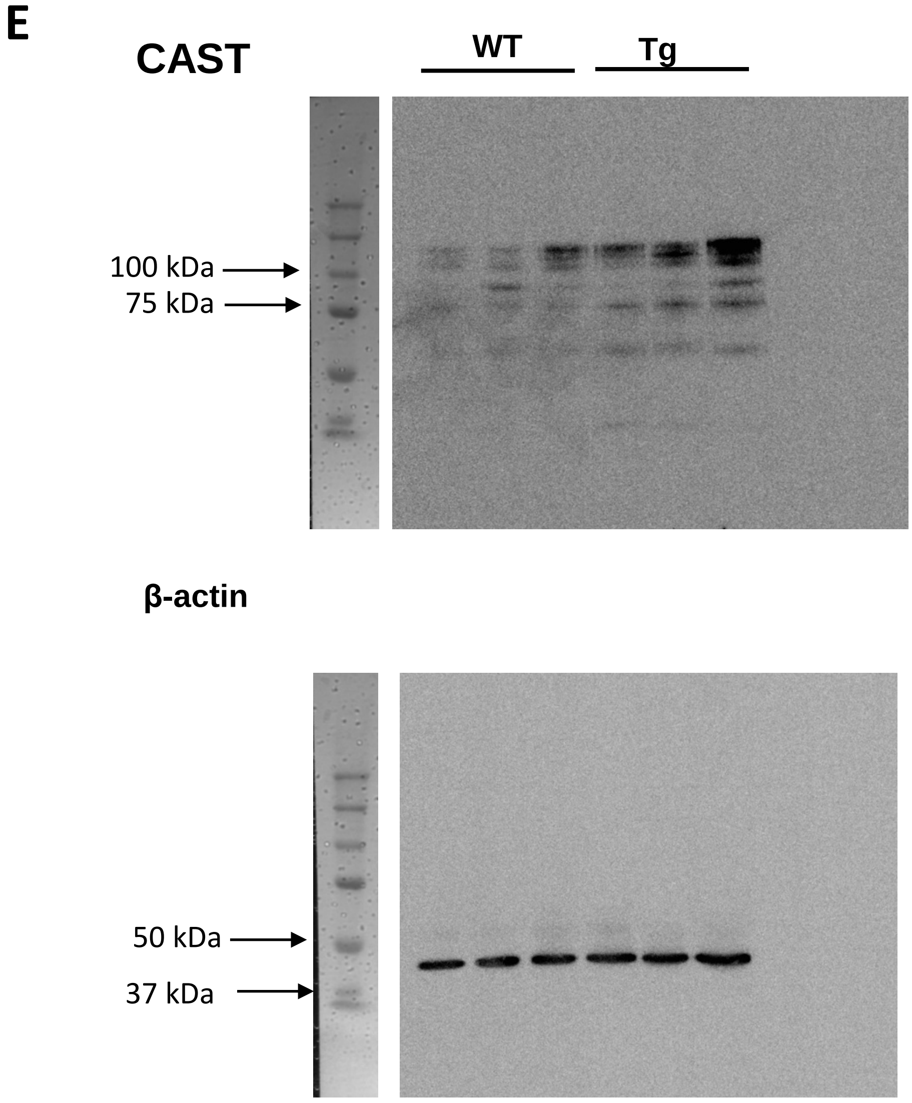

Figure 1

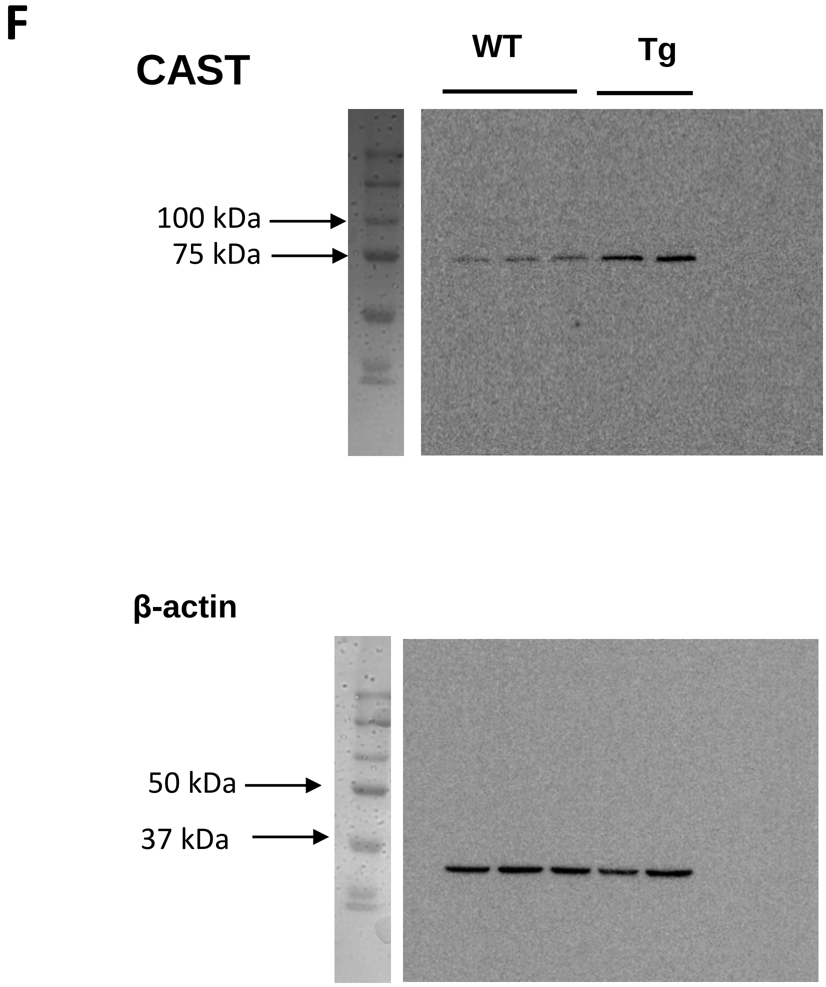

Figure 5

B

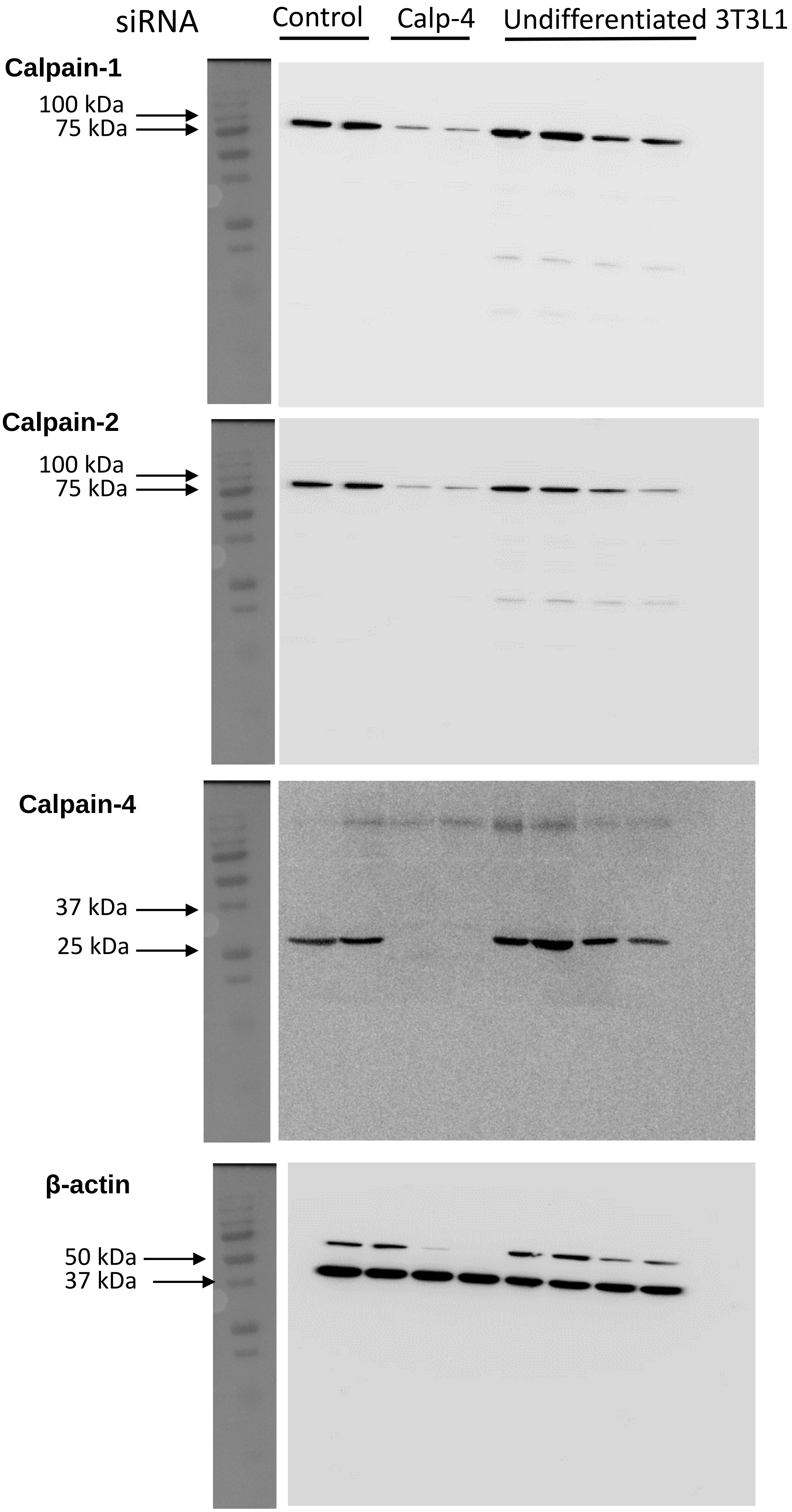
siRNA
Control
Calp-4
Undifferentiated 3T3L1
Calpain-1
100 kDa
75 kDa
Calpain-2
100 kDa
75 kDa
Calpain-4
37 kDa
25 kDa
β-actin
50 kDa
37 kDa

Figure 5

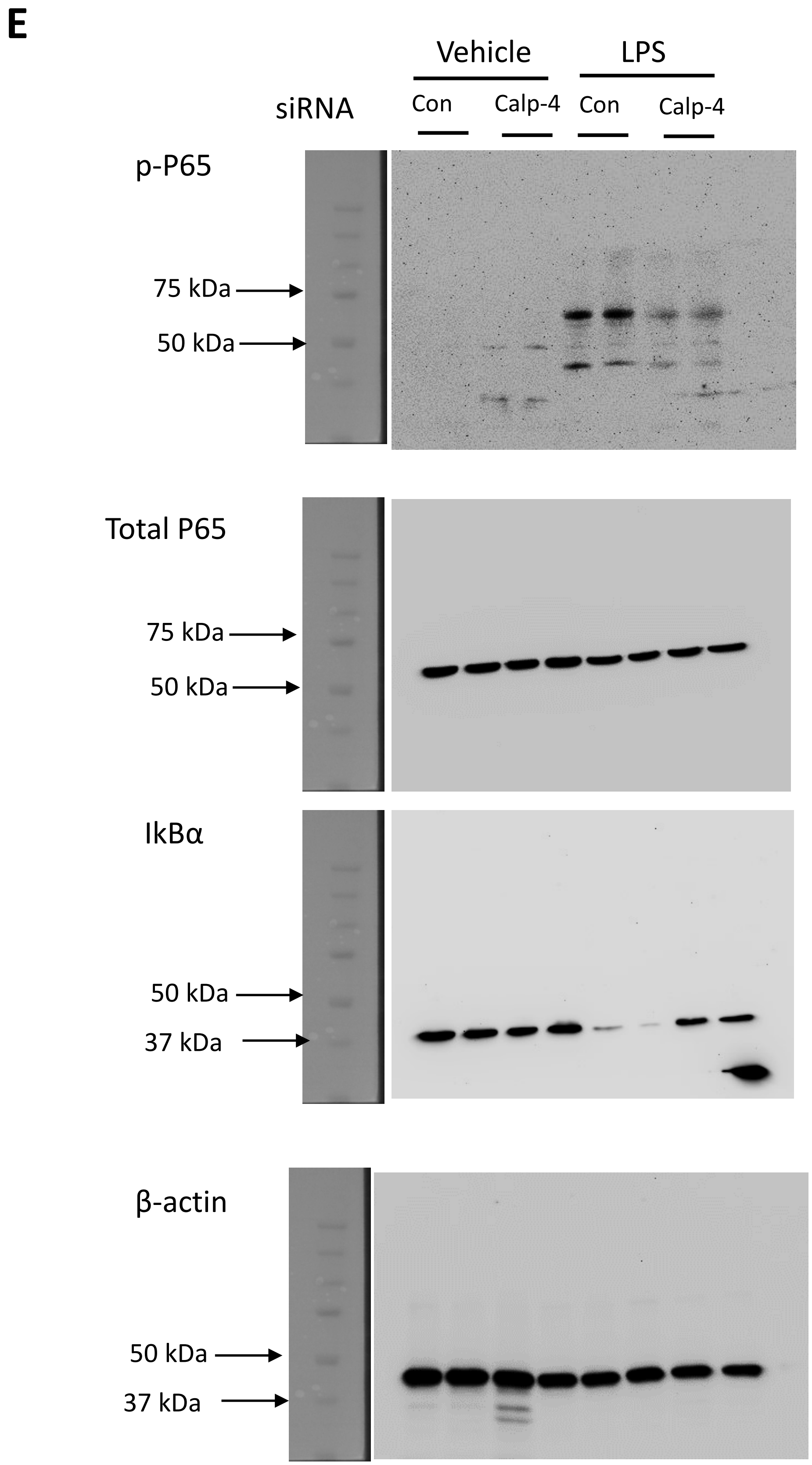
E
Vehicle
LPS
siRNA
Con
Calp-4
Con
Calp-4
p-P65
75 kDa
50 kDa
Total P65
75 kDa
50 kDa
IkBα
50 kDa
37 kDa
β-actin
50 kDa
37 kDa
